# Limited Visibility and Perception of the Clinical Relevance of Clopidogrel Pharmacogenetics in Cardiology Literature

**DOI:** 10.1111/cts.70584

**Published:** 2026-05-11

**Authors:** Cinzia Dello Russo, Luigi Venetucci, Abisope Akintola, Dimitri Gagliardi, Ronnie Ramlogan, Munir Pirmohamed

**Affiliations:** ^1^ Department of Pharmacology and Therapeutics, Institute of Systems, Molecular and Integrative Biology University of Liverpool Liverpool UK; ^2^ Department of Translational Medicine and Surgery, Section of Pharmacology Università Cattolica del Sacro Cuore ‐ Fondazione Policlinico Universitario A. Gemelli, IRCCS Rome Italy; ^3^ Division of Cardiovascular Sciences, Faculty of Biology, Medicine and Health The University of Manchester Manchester UK; ^4^ Manchester Institute of Innovation Research, Alliance Manchester Business School The University of Manchester Manchester UK; ^5^ The Wolfson Centre for Personalised Medicine, Centre for Drug Safety Science University of Liverpool Liverpool UK

**Keywords:** cardiovascular diseases, clopidogrel, CPIC guidelines, CYP2C19, DPWG guidelines, pharmacogenetics, UK CERSI‐PGx guidelines

## Abstract

Clopidogrel is an antiplatelet agent widely utilized in cardiology. It is a prodrug activated in the liver by the cytochrome P450 isoform CYP2C19. Variability in the *CYPC2C19* gene influences the activation and efficacy of clopidogrel. This is covered in guidelines from the Clinical Pharmacogenetics Implementation Consortium, the Dutch Pharmacogenetics Working Group, and more recently the UK Centre of Excellence in Regulatory Science and Innovation in Pharmacogenomics. Despite this extensive guidance, pharmacogenetic information is rarely used to guide clopidogrel prescription in cardiology patients. The present study assesses the visibility of the pharmacogenetic guidelines in the cardiology literature. We analyzed citations of the clopidogrel pharmacogenetic guidelines in the cardiology literature and in the cardiology guidelines/position statements on the treatment of acute coronary syndromes and stable coronary artery disease. Citations of these guidelines were found to be limited in the cardiology literature. Only 3 out of 19 cardiology guidelines/position statements refer to the pharmacogenetic guidelines. 58% of the cardiology guidelines/position statements mention clopidogrel pharmacogenetics but suggest that the use of pre‐emptive genotyping for *CYP2C19* variants to guide clopidogrel prescription is of limited clinical relevance. Genetically determined variation in clopidogrel efficacy has poor visibility in the cardiology literature and clinical guidelines. It is perceived to have limited benefits for clinical practice despite mounting evidence from randomized controlled trials and systematic reviews/meta‐analyses.

## Study Highlights

- What is the current knowledge on the topic?
  ○ Clopidogrel is an antiplatelet agent which requires a liver cytochrome P450 enzyme, CYP2C19, for metabolic activation. *CYP2C19*, the encoding gene, is polymorphic, with allelic variants that lead to reduced or no enzymatic activity with an impact on clopidogrel efficacy. Various pharmacogenetic guidelines have been developed to guide clopidogrel prescription based on genotype. Pharmacogenetics is included in clopidogrel drug labels by different regulators, although pre‐emptive *CYP2C19* pharmacogenetic testing is not mandated. In 2010, the FDA also included a warning box recommending to consider alternative treatments in CYP2C19 poor metabolisers.
- What question did this study address?
  ○ To address the extent to which pharmacogenetic prescribing guidelines are visible in the cardiology literature and in the major cardiology guidelines.
- What does this study add to our knowledge?
  ○ Pharmacogenetic guidelines for clopidogrel prescription are not visible to cardiologists through the international literature. Despite the high volume of publications related to clopidogrel in cardiovascular journals, citations of international pharmacogenetics guidelines are limited. We found no citations in major cardiology guidelines, and when reported, the pharmacogenetics of clopidogrel is perceived to be of limited usefulness.
- How might this change clinical pharmacology or translational science?
  ○ We suggest that to facilitate the implementation of pharmacogenetics in clinical cardiology, there is a need for greater pharmacogenetic education amongst cardiologists. This can be supported by identifying clinical champions and conducting feasibility studies. We also believe that regulators may play an important role by updating drug labels and/or issuing clear guidance (e.g., NICE recommendations on *CYP2C19* genotyping to guide clopidogrel prescription in stroke).

## 1 Introduction

Over the past 20 years, it has become increasingly clear that genetic variability can influence the efficacy and safety of drugs. Pharmacogenetics is the study of the effects of genetic variability on the response (efficacy and safety) to drugs [1]. Several evidence‐based pharmacogenetic prescribing guidelines are available to optimize the prescription of many drugs based on patient genetic background. These guidelines have been mainly issued by two international consortia in pharmacogenetics, the Clinical Pharmacogenetics Implementation Consortium (CPIC) (Supporting Information, Box 1) and the Dutch Pharmacogenetics Working Group (DPWG) (Supporting Information, Box 2). Despite extensive guidance, the adoption of a pharmacogenetic approach in clinical practice so far has been limited but has developed momentum recently [2].

Cardiology is a specialty with a good track record of rapid implementation of innovations, both of new technologies (e.g., stents, pacemakers and defibrillators) and new drugs (SGLT2 inhibitors, mavacamten and inclisiran). However, implementation of pharmacogenetics has been more limited. For instance, clopidogrel is a drug widely used in cardiology that has extensive pharmacogenetic guidance [3], but there is limited evidence of the uptake of pharmacogenetic‐guided prescribing into clinical practice. Clopidogrel is an oral antiplatelet drug indicated for the treatment of all forms of acute coronary syndrome (ACS), including unstable angina, non‐ST‐elevation and ST‐elevation myocardial infarction and stable coronary artery disease (CAD). In addition, clopidogrel is also used for the prevention of recurrent vascular events after a recent stroke or in patients with established peripheral arterial disease (PAD).

Clopidogrel is a prodrug that requires metabolism to form its active metabolite [4], which irreversibly bind to the P2Y12 receptor on platelets inhibiting aggregation. In vivo, approximately 85% of the drug is rapidly hydrolysed and inactivated by the hepatic enzyme, carboxylesterase 1 (CES1) [3, 5]. Activation of clopidogrel occurs in the liver, where the remaining 15% of the drug undergoes two sequential metabolic steps. These oxidative reactions lead to a first intermediate inactive metabolite (2‐oxo‐clopidogrel) and then to the active thiol metabolite (R‐130964) [3, 4, 5]. Despite different cytochrome P450 (CYP) enzymes, including CYP1A2, CYP2B6, CYP2C9, CYP2C19, and CYP3A4/5, being involved in the metabolism of clopidogrel, its activation is largely due to CYP2C19 [6, 7]. CYP2C19 enzyme is encoded by a polymorphic gene with several *CYP2C19* allelic variants that encode for proteins with variable enzymatic activity, e.g., no function or reduced function [5]. The combination of these variants can impact significantly on the metabolic activity of the CYP2C19 enzyme. Absent or reduced CYP2C19 metabolism impairs the activation of clopidogrel and decreases its pharmacological effects to varying extents [8]. CYP2C19*2 is the most frequent no function variant with an allelic frequency ranging from 10.4% in Latinos to 61.0% in the Oceanian population. The CYP2C19*3 variant is more frequent in the East Asian (7.2%) and Oceanian (14.6%) populations [9]. Interestingly, 57% of the UK South‐Asian ancestry population can be classified as CYP2C19 poor or intermediate metabolisers (PM or IM) largely because of the high frequency of the CYP2C19*2 allelic variant [10]. South Asians, accounting for 9% of the British population, are vulnerable to cardiovascular diseases [11] and have an increased risk of recurrent myocardial infarction when treated with clopidogrel because of the high frequency of the CYP2C19 PM and IM phenotypes [10]. The Hawaiian population is largely composed of Pacific Islanders and East Asians with a high allelic frequency of both CYP2C19*2 and *3 variants compared to Caucasians [12]. The CAPRIE clinical trial—which led to the approval of clopidogrel as an alternative therapy to aspirin for the secondary prevention of thrombotic events—studied 19,185 subjects over 3 years but 95% of the participants were Caucasians [13]. The lack of ethnic diversity in the clinical trial together with the lack of appreciation of the higher frequency of no function alleles in the Hawaiian population has led to a significantly higher rate of death in native Hawaiians (4.8%) in comparison to Caucasians (2.5%) [12], and a successful lawsuit against the manufacturers of clopidogrel [14].

Pharmacogenetically‐mediated variation in clopidogrel efficacy may have particular relevance in the elderly. Clopidogrel is the preferred option for the treatment of ACS (with and without PCI) and CAD in this cohort, with a recent scientific statement from the American Heart Association (AHA) on the management of ACS in the elderly stating [15]: “*In the older patient with ACS, clopidogrel is the preferred P2Y12 inhibitor because of a significantly lower bleeding profile than ticagrelor or prasugrel, but for patients with STEMI or complex anatomy, the use of ticagrelor is reasonable*”. A recent study has clearly demonstrated that elderly patients with no function alleles of *CYP2C19* undergoing PCI experience a substantially higher rate of major adverse cardiac events in the 3 years following the PCI [16].

Overall, clopidogrel remains a widely prescribed drug in the cardiovascular setting both in Europe and North America [17, 18], especially in patients at higher risk of bleeding [19] and in the elderly (as mentioned above) due to the more favorable safety profile compared to prasugrel and ticagrelor. Without testing for the *CYP2C19* allelic variants, there is an increased risk of therapeutic failure for patients with impaired metabolism. While the drug labels across different regulators report pharmacogenetic information, they do not mandate pre‐emptive pharmacogenetic testing for *CYP2C19*. In March 2010, the US Food and Drug Administration (FDA) approved drug label for Plavix, the branded medicinal product containing clopidogrel, was updated to include a warning box highlighting the reduced efficacy of clopidogrel in CYP2C19 poor metabolisers and recommending to ‘*Consider alternative treatment or treatment strategies in patients identified as CYP2C19 poor metabolizers*’. The same recommendation is reported in the last available FDA approved drug label [20].

Both CPIC and DPWG have provided several pharmacogenetic guidelines to optimize the prescribing of clopidogrel. The first CPIC pharmacogenetic prescribing guideline was published in 2011 [21], followed by two updates in 2013 [22] and 2022 [8]. The last update was deemed necessary to include results from prospective randomized clinical trials (RCTs), multicentre implementation studies, and meta‐analyses of *CYP2C19* genotype‐guided antiplatelet therapy [23, 24] demonstrating the clinical utility of *CYP2C19* genotyping before the initiation of clopidogrel therapy. Similarly, the DPWG published a pharmacogenetic‐based guideline for clopidogrel in 2011 [25], with further updates available via the ClinPGx database [9]. Despite the availability of pharmacogenetic prescribing guidelines, pharmacogenetic testing is rarely performed before prescribing clopidogrel.

In order to investigate the significance of why this may be so, we have analyzed the citations of the CPIC guidelines in (a) the general literature and (b) in the cardiology guidelines/position statements in relation to the treatment of ACS and stable CAD issued by major international cardiovascular societies including the European Society of Cardiology (ESC), the AHA, and the American College of Cardiology (ACC).

## 2 Methods

The impact of the CPIC clopidogrel guidelines released in 2013 [22] and 2022 [8] was evaluated by analyzing the citation patterns in cardiovascular journals indexed in PubMed. We focused on the CPIC guidelines given they are readily available (https://cpicpgx.org/). We limited the analysis to the two most recent guidelines as they have the highest number of citations per year after publication. We extracted the list of articles, without differentiating based on the type of paper, citing the two CPIC clopidogrel guidelines by accessing the PubMed database on 24 November 2025. The articles were classified by the citing journals into 7 categories: 1. Pharmacology/Pharmacy; 2. Cardiovascular; 3. Neurology/Neuroscience; 4. General Medicine; 5. Genetics; 6. Basic Science; 7. Other. The full list of the articles citing these guidelines is provided in the Supporting Information, Data S1 and Data S2.

To account for differences in publication volume across journal categories, we performed a normalized citation analysis on the 2013 CPIC guidelines. For each journal category, we calculated a normalized 2013 CPIC citation ratio, defined as the number of identified citations to the 2013 CPIC guideline divided by the number of clopidogrel‐related publications indexed in the PubMed database in the corresponding reference journal set. Clopidogrel publication volume was determined using a 2013–2025 clopidogrel publication dataset. Overall, this approach provided a journal‐category‐specific citation metric weighted to clopidogrel publication output.

To gain deeper insights into the visibility of clopidogrel pharmacogenetics amongst the cardiology scientific community, we analyzed how frequently clopidogrel pharmacogenetics was included in randomized clinical trials assessing outcomes after clopidogrel administration. This was done by extracting data from the clinicaltrials.gov database (https://clinicaltrials.gov/) using two separate searches with duplicates identified and excluded:

Search 1: The term acute coronary syndrome was used for the condition/disease and clopidogrel as the intervention/treatment within a specific time window (01/01/2011–25/11/2025). Subsequently, we included CYP2C19 in the ‘other term’ box to search for trials related to clopidogrel pharmacogenetics.

Search 2: The terms coronary artery disease and clopidogrel were used within the same time window, from 2011 to 2025, with CYP2C19 added as an additional search term.

In addition, we reviewed the guidelines and position statements released by the ESC and the AHA/ACC regarding the management of coronary interventions and coronary artery disease to establish whether the CPIC and the DPWG guidelines were acknowledged. We searched PubMed, the ACC website and the ESC website for guidelines on the diagnoses and management of stable CAD and ACS (particularly for ST‐elevation myocardial infarction, non‐ST‐elevation myocardial infarction) and coronary revascularization. We also extracted data from the position statements on the use of antiplatelet medications. We limited our analysis to guidelines and position statements published since 2011, the year of the first publication of the pharmacogenetic guidelines. The full list of these documents is provided in the Supporting Information.

We performed a textual analysis to establish whether any of these guidelines/position statements mentioned clopidogrel pharmacogenetics and whether they expressed an opinion on the clinical relevance. In addition, we analyzed the areas of expertise amongst the guidelines' authors by identifying their main specialities from publicly available curricula and evaluating their involvement in clopidogrel pharmacogenomics through an assessment of their publication track records.

## 3 Results

### 3.1 What Do the CPIC and DPWG Guidelines Say?

Recommendations from the 2022 CPIC guideline [8] for clopidogrel use in cardiovascular disease are summarized in Table 1. It is important to highlight that based on the level of evidence (Supporting Information, Box 1), the recommendation for cardiovascular diseases is classified as strong. The summary of the 2018 DPWG guideline [9] is presented in Table 2. The DPWG considers the association between *CYP2C19* and clopidogrel as clinically relevant, assigning the pharmacogenetic test a clinical implication score of 8+. This means that the pharmacogenetic test is considered essential before starting clopidogrel therapy (Supporting Information, Box 2).

**TABLE 1:** CPIC recommendations adapted from the 2022 CPIC guidelines [8].

Genotype	Recommendation	Classification of recommendation – ACS and/or PCI
CYP2C19 ultrarapid metabolizer	Use standard dose of clopidogrel	Strong
CYP2C19 rapid metabolizer	Use standard dose of clopidogrel	Strong
CYP2C19 normal metabolizer	Use standard dose of clopidogrel	Strong
CYP2C19 likely intermediate metabolizer	Avoid standard dose of clopidogrel Use prasugrel or ticagrelor	Strong
CYP2C19 intermediate metabolizer	Avoid standard dose of clopidogrel Use prasugrel or ticagrelor	Strong
CYP2C19 likely poor metabolizer	Avoid clopidogrel Use prasugrel or ticagrelor	Strong
CYP2C19 poor metabolizer	Avoid clopidogrel Use prasugrel or ticagrelor	Strong

**TABLE 2:** Summary of DPWG guidelines November 2018 update [9].

Genotype	Recommendation
CYP2C19 poor metabolizer	Avoid clopidogrel, use prasugrel or ticagrelor
CYP2C19 intermediate metabolizer	Choose an alternative or double the dose to 150 mg/day (600 mg loading dose).
CYP2C19 ultrarapid metabolizer	No action is required

### 3.2 Are the Clopidogrel Pharmacogenetic Guidelines Visible in the Cardiology Literature?

**FIGURE 1:**
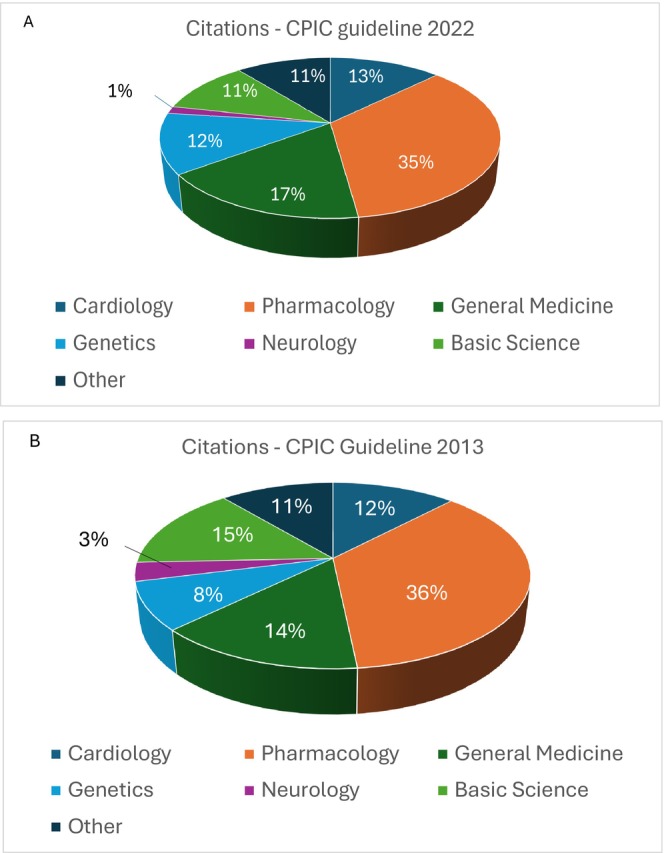
Citation analysis of the CPIC 2022 (A) and 2013 (B) guidelines by Medical Sub‐disciplines. The lists of articles citing the two CPIC guidelines were extracted from PubMed on the 24 November 2025. Articles were categorized in different groups according to the main field of Medicine they referred to, as indicated in the Figure legend in each chart. The percentage of articles in each group is indicated, considering a total citation number of 205 for the CPIC 2022 guideline and 398 for the CPIC 2013 guideline.

The citation analysis of the CPIC guideline from 2022 [8] showed that this guideline had received 205 citations in 103 journals. As shown in Figure 1A, most of the citations (*n* = 72, 35%) were in pharmacology journals. There were 35 citations (17%) in general medicine journals, but only 26 citations (13%) in cardiovascular journals. Of these, only 3 were found amongst the five major journals in which cardiovascular guidelines/position statements are most frequently published: Nature Review Cardiology (0 citations), European Heart Journal (0 citations), Circulation (1 citation), Journal of the American College of Cardiology (2 citations), and JAMA Cardiology (0 citations).

The analysis of the CPIC guideline from 2013 revealed a similar trend (Figure 1B). Of the 398 citations (from 191 journals listed in PubMed), pharmacology (*n* = 143, 36%) and general medicine (*n* = 57, 14%) journals represented the main citing journals. A total of 49 citations (12%) were in cardiovascular journals, but only 4 were in one of the five major cardiovascular journals (2 in Nature Reviews Cardiology, 1 in Circulation and 1 in JAMA Cardiology). These data point to the limited visibility of clopidogrel pharmacogenetic guidelines in major cardiovascular journals.

A normalized citation analysis on the 2013 CPIC guidelines showed that cardiology journals remained the category with the lowest 2013 CPIC citation ratio amongst the major journal categories examined (Table 3). The normalized citation ratio was 3.81% (49/1285) for cardiology, compared with 23.40% (143/611) for pharmacology, 10.71% (57/532) for general medicine, 20.70% (59/285) for basic science, 16.06% (44/274) for other journals, 7.10% (13/183) for neurology journals, and 68.75% (33/48) for genetics journals. Overall, the total normalized ratio was 12.37% (398/3218).

**TABLE 3:** Normalized 2013 CPIC citation ratio by medical specialty in years 2013–2025.

Rank	Specialty	Citations of the 2013 CPIC guideline (*n*)	Clopidogrel papers indexed In PubMed in 2013–2025 (*n*)	Normalized 2013 CPIC citation ratio
1	Genetics	33	48	0.6875
2	Pharmacology	143	611	0.2340
3	Basic science	59	285	0.2070
4	Other	44	274	0.1606
5	General Medicine	57	532	0.1071
6	Neurology	13	183	0.0710
7	Cardiology	49	1285	0.0381
	TOTAL	398	3218	0.1237

*Note:* Normalized 2013 CPIC citation ratio = 2013 CPIC number of citations per specialty ÷ number of clopidogrel publications indexed in the PubMed database in years 2013–2025 per specialty. A higher ratio indicates that the specialty's journals contribute more 2013 CPIC citations relative to clopidogrel publication volume.

The impact of the DPWG prescribing guidelines in the cardiovascular literature could not be directly addressed. The first DPWG guideline published in 2011 [25] includes recommendations for several drug‐gene pairs, but the recent updates have not been published in the international literature.

To gain further insights into the inclusion of clopidogrel pharmacogenetics during clinical research development, we examined whether CYP2C19 was mentioned in clinical trials of clopidogrel. We found a total of 418 trials studying clopidogrel in the setting of ACS and/or CAD and registered in the clinicaltrials.gov database between 1 January 2011 and 25 November 2025. Amongst those, only 42 (10%) trials mentioned CYP2C19. This estimate may be conservative considering that not all the details of the trials are always reported in the database.

### 3.3 Are CPIC and DPWG Guidelines Cited by the ESC and AHA/ACC Guidelines on the Treatment of CAD and ACS? Do These Guidelines Mention the Pharmacogenetics of Clopidogrel?

We identified a total of 14 guidelines and 5 position/consensus statements, which are listed in Supporting Information, Table S1 (ESC) and Table S2 (ACC/AHA). None of the cardiovascular guidelines cited the pharmacogenetics guidelines, and although 3 out of the 5 position statements cited the CPIC guidelines, none referred to the DPWG guidelines.

We then performed a more detailed textual analysis of these 19 documents to understand whether they mentioned that the efficacy of clopidogrel may be influenced by pharmacogenetic factors. Eleven of 19 documents (58%) mentioned the issue of clopidogrel pharmacogenetics (Tables S1 and S2). The comments provided by each document are summarized in the Supporting Information, Tables S3 (ESC) and S4 (AHA/ACC) and show a clear evolution of the thinking of the interventional cardiology community. The guidelines refer mainly to the use of *CYP2C19* testing to guide the prescribing of clopidogrel for de‐escalation from more potent antiplatelet treatments (ticagrelor and prasugrel) in order to reduce the risk of bleeding. The earlier guidelines, until they were superseded in 2020, stated that there was no clear evidence to support the use of *CYP2C19* testing to guide clopidogrel prescribing and that randomized controlled trials were needed to provide robust evidence.

The more recent guidelines, published since 2020, show a different approach between North America and Europe. The North American AHA/ACC guidelines have omitted the issue of clopidogrel pharmacogenetics (Table S2, numbers 6, 7 and 11). This includes the recent 2025 ACC/AHA guidelines on the management of ACS (Table S2, number 11). However, this topic has been addressed in three expert consensus statements (Table S2, numbers 8, 9 and 10). The AHA/ACC consensus report on antiplatelet treatment in patients with CAD, published in 2023 (Table S2, number 8), acknowledges the publication of the POPular Genetics trial [23] which showed non‐inferiority of *CYP2C19* testing guided use of clopidogrel and a lower rate of bleeding compared to the use of ticagrelor or prasugrel. However, the consensus statement calls for more data (probably from randomized controlled trials) to support the adoption of pharmacogenetic testing to guide clopidogrel prescription (Table S4, number 8). The recent scientific statement from the AHA (Table S4, number 9), however, provides a comprehensive review of the literature and concludes that:

“A precision medicine approach based on CYP2C19 genetic testing results in which LOF carriers are prescribed ticagrelor or prasugrel and noncarriers are prescribed clopidogrel decreases the risk of ischemic events compared with universal clopidogrel and decreases the risk of bleeding compared with universal ticagrelor or prasugrel and thus may offer a more balanced therapeutic approach” (page e141).

In November 2024, a further update on the use of genetic testing in PCI was published in JACC Cardiovascular Intervention (Table S2, number 10). Variability in the response to clopidogrel is acknowledged and the update recognized that genetic testing may guide selection of antiplatelet agents. However, the authors argue that non‐uniform results of randomized controlled trials have led to very limited recommendations from major guidelines to implement this strategy in patients undergoing PCI. They suggest that this strategy may be utilized in specific cases (especially when there is a perception of a high risk of bleeding) and call for more randomized controlled trials (Table S4, number 10). Thus far, the only document that provides a clear endorsement on the use of *CYP2C19* testing to guide the prescription of clopidogrel is the 2024 Scientific Statement from the AHA (Table S4, number 9), i.e., pre‐emptive *CYP2C19* testing can be beneficial. It is worth mentioning that this statement was endorsed only by the AHA and was promoted mainly by the AHA Council on Genomic and Precision Medicine which advocates for the use of genetic data in clinical practice.

The ESC guidelines published since 2020 (Table S1, numbers 6, 7, and 8) report that recent randomized controlled trials [23] and meta‐analyses [24] have shown that the use of genetic testing for clopidogrel is non‐inferior to the standard approach (use of ticagrelor or prasugrel) and causes a lower rate of bleeding. However, these guidelines do not endorse routine use of genetic testing and suggest that this may be considered in some cases if there is “a perception” of an increased risk of bleeding (Table S3, numbers 6, 7 and 8). The 2020 ESC Guidelines for the management of ACS in patients presenting without persistent ST‐segment elevation (Table S3, number 6) provide a weak recommendation (IIb). Similarly, the 2023 ESC Guidelines for the management of acute coronary syndromes and the 2024 ESC Guidelines for the management of chronic coronary syndromes (Table S3, numbers 7 and 8) provide a weak recommendation for *CYP2C19* testing (classed as IIb). This type of recommendation suggests that the usefulness/efficacy of such testing is less well established according to either evidence and/or opinion.

From the analysis of the areas of expertise of the authors involved in the writing of the ESC and the AHA/ACC guidelines (Tables S5 and S6), it emerged clearly that these guidelines are developed within the cardiology community, without a relevant involvement of other experts, including, for example, pharmacologists and geneticists. This trend seems to change for the latest documents published by the North American associations (Table S6). On the other hand, it appears that there is awareness of clopidogrel pharmacogenetics and variability in its efficacy amongst the authors, as documented by their publication track records (Tables S5 and S6).

## 4 Discussion

Clopidogrel provides an excellent case study to analyze the factors influencing the adoption of pharmacogenetics in cardiology. There is clear evidence of the role of genetic variability in activating clopidogrel which may compromise its efficacy [18]. This has led to the development of guidelines by both CPIC and DWPG on the utilization of genetic testing to guide the prescription of clopidogrel. Specifically, these guidelines aim to inform drug prescription when pharmacogenetic data are available, with DWPG having recently introduced clinical implication scores referring to the clinical utility of pharmacogenetic testing before drug administration (Supporting Information, Box 2). However, despite these guidelines and accumulating evidence [23, 24], the uptake of clopidogrel pharmacogenetics into clinical practice has been low. The findings of our paper highlight the discrepancy in the enthusiasm for clopidogrel pharmacogenetic testing between the cardiology and non‐cardiology literature:

- There are few citations of the CPIC guidelines in the main cardiology journals and differences in publication volume alone do not explain the lower representation of the pharmacogenetics guidelines within the cardiology literature;
- Between 2011 and 2025, a limited number of trials testing clopidogrel in the setting of acute coronary syndrome and coronary artery disease included the term ‘CYP2C19’.
- Out of 14 guidelines and 5 consensus statements from ESC and AHA/ACC, only three cite the CPIC guidelines and none the DWPG guidelines;
- 58% of the guidelines and consensus statements mention clopidogrel pharmacogenetics (Tables S1 and S2) but only the 2024 AHA statement (Tables S2 and S4, number 9) endorses the use of genetic testing to guide the prescription of clopidogrel; and
- Pharmacogenetics of clopidogrel is perceived to be of limited usefulness by cardiologists.

### 4.1 Why Is There Limited Visibility of Clopidogrel Pharmacogenetics in the Cardiology Literature?

Over the last 15 years, the pharmacogenomic community has provided clear and detailed guidance to embed pharmacogenetics into clinical practice. The clopidogrel guidelines clearly state that the level of evidence supporting the use of genotyping to guide clopidogrel prescribing in cardiology is very strong, but these guidelines have limited visibility in the mainstream cardiovascular literature.

In cardiology, international guidelines are one of the main drivers of the diffusion of new knowledge, adoption of change, and improvement of patient care [26, 27]; therefore, the lack of citations of pharmacogenomic guidelines in the mainstream cardiology guidelines can be considered a major barrier to the diffusion of such knowledge. This has also translated into limited consideration of clopidogrel pharmacogenetics in recent clinical trials. This is acknowledged by the recent statement from the AHA (Tables S2 and S4, number 9), which calls for the integration of recently published evidence on clopidogrel pharmacogenetics into cardiology clinical guidelines.

A possible reason why pharmacogenetic guidelines are not on the radar of cardiologists may be the limited communication between clinical pharmacology and cardiology. The two specialities seem to be working in independent silos [28, 29] each developing their own innovation pathways, probably related to the ultra‐specialized nature of modern medicine. Another reason is the representativeness of the international cardiology guideline committees, which hitherto have not included experts in genetics and pharmacology (Tables S5 and S6). Similarly, pharmacogenetic guideline committees lack sufficient representation from clinical cardiology experts among their authors.

### 4.2 Why Is Clopidogrel Testing Perceived To Be of Limited Usefulness by the Cardiology Community?

There is no single simple answer to this question. Two main factors deserve some consideration which may explain, at least partially, poor adoption.

First is ‘familiarity’. In healthcare, prescribing a drug or using a method for a long time may lead to therapeutic inertia, where practitioners prefer to continue with their familiar prescribing pathway rather than disrupting it by introducing a new or unfamiliar technology. This is clearly exemplified by the 2020 ESC guidelines (Table S1, number 6) on the management of non‐ST‐elevation myocardial infarction (NSTEMI). The guidelines attribute a IIb recommendation level for the use of genetic testing to guide clopidogrel prescription despite the high level of evidence of clinical utility (classified as A). By contrast, the same guidelines give a higher level of recommendation (IIa) to the long‐term use of beta‐blockers in patients with normal cardiac function despite the lower level of evidence in support of their adoption. The level of evidence for beta‐blockers, amongst the most widely used drugs by cardiologists, is indeed B because of the conflicting literature evidence [30, 31]. On this premise, it is not surprising that many cardiologists would find it easier to recommend the use of beta‐blockers (a familiar drug) rather than endorse the adoption of *CYP2C19* testing before prescribing clopidogrel. This may be due to limited familiarity with pharmacogenetic testing, as genetic testing is not in widespread use in cardiology. Within cardiology, genetic testing is utilized only in the management of inherited cardiac conditions [32]. However, even in this area, genetic testing is mainly controlled by geneticists and results normally take several weeks to become available. Interpretation of the data is complex with a significant number of variants of uncertain significance. It is possible that the very niche use, long turnaround time to obtain results and the complicated interpretation may have generated skepticism, especially in other sub‐specialties like interventional cardiology, where the ability to make rapid and clear‐cut decisions is very important.

Overcoming the limited uptake may be facilitated by the presence of champions acting (1) as advocates for adoption, and (2) providing the relevant expertise and knowledge to facilitate implementation [33]. In this respect, it is important to mention that implementation of various innovations in cardiovascular medicine have been facilitated by industry. There is evidence of growing involvement of industry in cardiovascular pharmacogenetics [34] (e.g., the development of point of care testing) or with the use of mavacamten in the UK [35].

A second factor underlying the perception of limited clinical relevance of clopidogrel pharmacology could be linked to the advent of new technologies over the last 15 years. For example, first generation drug eluting stents were associated with high rates of in‐stent thrombosis mainly due to poor stent endothelization [36, 37]. The development of second‐generation drug eluting stents (with thinner struts, biocompatible drug eluting polymers and more selective antiproliferative medications) substantially improved stent endothelization and reduced the rate of in‐stent thrombosis [37].

Furthermore, advances in intravascular imaging techniques have aided the reduction in thrombosis derived from stent malapposition [38]. The development of intravascular imaging techniques such as intravascular ultrasound [39] and optical coherence [39] tomography has enabled a more accurate assessment of stent deployment. This, in turn, has resulted in a significant reduction in the rate of stent thrombosis. Such developments have made cardiologists less concerned about in‐stent thrombosis and the efficacy of antiplatelet medications and may have led to reduced interest in the pharmacogenetics of clopidogrel.

### 4.3 How Can Visibility and Information Be Improved?

We have highlighted that clopidogrel pharmacogenetic guidelines are not very visible to the cardiology community. To increase awareness amongst cardiologists and clinical practitioners, pharmacogenetics should be introduced via traditional channels of education. A complementary pathway for knowledge diffusion could be provided by the Regulators. A first step may include updating the drug label. This has been shown to be effective in the use of dihydropyrimidine dehydrogenase (DPD) testing prior to treatment with fluoropyrimidines [40], which prompted the implementation of pharmacogenetic testing across Europe [41] and in the UK [42].

Recently, NICE in the UK has recommended the use of *CYP2C19* genotyping prior to the prescribing of clopidogrel for patients with stroke or TIA [43]. Genotyping for the same gene is also mandated by the Medicine & Healthcare products Regulatory Agency (MHRA) for the prescription of mavacamten for the treatment of hypertrophic obstructive cardiomyopathy [44]. This does lead to a conundrum for the cardiology community. While *CYP2C19* pharmacogenetic testing has been implemented for the prescription of mavacamten in the NHS [35], the same test cannot be ordered to guide the prescription of clopidogrel for coronary artery disease because it is not reimbursed. A recent development is the publication of a guideline developed by the UK Centre of Excellence in Regulatory Science and Innovation in Pharmacogenomics (CERSI‐PGx) [45] on the use of pharmacogenetic testing for *CYP2C19* which recommends pre‐emptive genotyping for all patients undergoing clopidogrel therapy regardless of the underlying indications [46]. Importantly, the committee that developed this guideline was representative of the different specialties that need to consider the use of clopidogrel.

To facilitate the adoption of *CYP2C19* testing in clinical practice, there is a need to demonstrate that such methods are not complex and can lead to rapid and informed decisions. The work performed in the Netherlands by ten Berg's Group [2, 47] provides an excellent example of efficient and impactful implementation of *CYP2C19* testing in clinical practice. Using a point of care test that provides information within an hour, they demonstrated that the use of clopidogrel guided by pharmacogenetic testing was non‐inferior to the standard approach in preventing ischaemic events but also led to less bleeding. This also resulted in significant savings, representing an excellent blueprint for implementation in other countries.

## 5 Limitations of Our Study

Our study has several limitations: (i) the citation analysis focused solely on the 2013 and 2022 CPIC guidelines and did not capture the direct influence of DPWG or earlier publications; (ii) the review of guidelines focused on major Western societies (mainly North America and Europe/UK), potentially missing perspectives from regions where *CYP2C19* loss‐of‐function variants are more prevalent; (iii) the study relied on indirect proxies for professional perception, and this cannot fully substitute for direct engagement through surveys or interviews. To our knowledge, there have been no published surveys on the prescribing attitude towards pharmacogenomics of cardiologists in the UK. A recent report on personalized prescribing by the UK Royal College of Physicians and British Pharmacological Society identified awareness by healthcare professionals as a barrier to implementation [48]. Our study, by documenting a gap between robust pharmacogenetic evidence and its limited clinical uptake in the cardiology community, provides the groundwork for future implementation studies and targeted educational interventions.

In conclusion, based on an integrated bibliometric data search with a qualitative review of guideline documentation, we have shown that clopidogrel pharmacogenetics has limited visibility in the cardiology literature and relevant clinical guidelines implicitly consider it of limited clinical utility. Our study highlights the importance of education of cardiologists and allied healthcare professionals to enhance awareness of pharmacogenetics and its clinical benefits [49]. There is also a clear need for implementation projects to demystify the concept of pharmacogenetics, presenting it as a useful tool to optimize medical care [2].

## Author Contributions

All authors wrote the manuscript. D.G., R.R., and M.P. designed the research. L.V. and C.D.R. performed the research. L.V., C.D.R., A.A., D.G., and R.R. analyzed the data.

## Funding

This work was supported by The Economic and Social Research Council Grant (ES/W011484/1).

## Conflicts of Interest

Munir Pirmohamed currently receives partnership funding, paid to the University of Liverpool, for the MRC Medicines Development Fellowship Scheme (co‐funded by MRC and GSK, AZ, Optum and Hammersmith Medicines Research). He has developed an HLA genotyping panel with MC Diagnostics but does not benefit financially from this. He is part of the IMI Consortium ARDAT (www.ardat.org). None of these funding sources have been used for the current paper. All other authors declared no competing interests for this work.

## Supporting information


**Box 1:** The Clinical Pharmacogenetics Implementation Consortium.
**Box 2:** The Dutch Pharmacogenetics Working Group.
**Table S1:** Inclusion of clopidogrel pharmacogenetics in the ESC guidelines and position statements published since 2011.
**Table S2:** Inclusion of clopidogrel pharmacogenetics in the ACC/AHA guidelines and position statements published since 2011.
**Table S3:** Textual analysis carried out on the ESC guidelines and position statements that mentioned clopidogrel pharmacogenetics. In column 1, the same number used in Table S1 is included.
**Table S4:** Textual analysis carried out on the ACC/AHA guidelines and position statements that mentioned clopidogrel pharmacogenetics. In column 1, the same number used in Table S2 is included.
**Table S5:** Analysis of the areas of expertise and involvement in clopidogrel pharmacogenomics amongst the authors of the ESC guidelines.
**Table S6:** Analysis of the areas of expertise and involvement in clopidogrel pharmacogenomics amongst the authors of the ACC/AHA guidelines.
Supplementary References.

**Data S1:** cts70584‐sup‐0002‐DataS1.pdf.

**Data S2:** cts70584‐sup‐0003‐DataS2.pdf.
