# Supplementary material for: Limited Visibility and Perception of the Clinical Relevance of Clopidogrel Pharmacogenetics in Cardiology Literature
Source: Clin Transl Sci. 2026 May 11;19(5):e70584. doi: 10.1111/cts.70584 (PMC13160923; doi:10.1111/cts.70584)
Supplement: Supplementary file 1 — Box 1: The Clinical Pharmacogenetics Implementation Consortium. Box 2: The Dutch Pharmacogenetics Working Group. Table S1: Inclusion of clopidogrel pharmacogenetics in the ESC guidelines and position statements published since 2011. Table S2: Inclusion of clopidogrel pharmacogenetics in the ACC/AHA guidelines and position statements published since 2011. Table S3: Textual analysis carried out on the ESC guidelines and position statements that mentioned clopidogrel pharmacogenetics. In column 1, the same number used in Table S1 is included. Table S4: Textual analysis carried out on the ACC/AHA guidelines and position statements that mentioned clopidogrel pharmacogenetics. In column 1, the same number used in Table S2 is included. Table S5: Analysis of the areas of expertise and involvement in clopidogrel pharmacogenomics amongst the authors of the ESC guidelines. Table S6: Analysis of the areas of expertise and involvement in clopidogrel pharmacogenomics amongst the authors of the ACC/AHA guidelines. Supplementary References. [file CTS-19-e70584-s002.docx]

**Limited visibility and perception of the clinical relevance of clopidogrel pharmacogenetics in cardiology literature**

Cinzia Dello Russo MD PhD^1,2§^ (<https://orcid.org/0000-0002-2538-3832>), Luigi Venetucci MD PhD^3§*^ (<https://orcid.org/0000-0002-8236-3993>), Abisope Akintola PhD^4^ (<https://orcid.org/0009-0006-9163-8537>), Dimitri Gagliardi PhD^4^ (<https://orcid.org/0000-0001-6916-5610>), Ronnie Ramlogan PhD^4^, Munir Pirmohamed MD PhD^2,5^ (<https://orcid.org/0000-0002-7534-7266>).

^1^Department of Pharmacology and Therapeutics, Institute of Systems, Molecular and Integrative Biology, University of Liverpool, Liverpool, United Kingdom.

^2^Department of Translational Medicine and Surgery, Section of Pharmacology, Università Cattolica del Sacro Cuore - Fondazione Policlinico Universitario A. Gemelli, IRCCS, Rome, Italy

^3^Division of Cardiovascular Sciences, Faculty of Biology, Medicine and Health, The University of Manchester, Manchester, United Kingdom.

^4^Manchester Institute of Innovation Research, Alliance Manchester Business School, The University of Manchester, Manchester, United Kingdom.

^5^The Wolfson Centre for Personalised Medicine, Centre for Drug Safety Science, University of Liverpool, Liverpool, United Kingdom.

^§^Luigi Venetucci and Cinzia Dello Russo equally contributed to the study as first authors.

* ***To whom the correspondence should be addressed:*** Dr Luigi Venetucci, Division of Cardiovascular Sciences, The University of Manchester, Core Technology Facility, 46 Grafton Street, Manchester, M139NT, United Kingdom. Email: [luigi.venetucci@manchester.ac.uk](mailto:luigi.venetucci@manchester.ac.uk)

**Box. 1 The Clinical Pharmacogenetics Implementation Consortium.**

The Clinical Pharmacogenetics Implementation Consortium (CPIC) is an international consortium interested in facilitating the use of pharmacogenetic tests for patients’ care. It provides detailed gene/drug clinical practice guidelines to enable the use of genomic biomarkers in clinical practice. Recommendations are based on systematic literature reviews and are periodically updated. Notably, the CPIC guidelines do not provide indications on when to perform a specific pharmacogenetic test (eligibility criteria) but only advise on the use of genetic information when available. Each guideline includes a grading of the level of evidence linking a genotype to a specific pharmacological phenotype, based on the quality of the studies. As explained in the guidelines, ‘*when evidence includes consistent results from well-designed, well-conducted studies’*, the level of evidence is graded high; ‘*when evidence is sufficient to determine effects, but the strength of the evidence is limited by the number, quality or consistency of the individual studies, generalizability to routine practice, or the indirect nature of the evidence’*, the level of evidence is graded moderate; ‘*when evidence is insufficient to assess the effects on health outcomes because of limited number or power of studies, important flaws in their design or conduct, gaps in the chain of evidence, or lack of information’*, the level of evidence is graded weak. Consequently, the strength of the recommendations is defined strong, ‘*when the evidence is high quality, and the desirable effects clearly outweigh the undesirable effects’*; moderate, *when there is a close or uncertain balance as to whether the evidence is high quality and the desirable effects clearly outweigh the undesirable effects’*; optional, ‘*when the desirable effects are closely balanced with undesirable effects, or the evidence is weak or based on extrapolations*. *In this case,* *there is room for differences in opinion as to the need for the recommended course of action’*. Finally, if there is ‘*insufficient evidence, confidence, or agreement to provide a recommendation to guide clinical practice at the time of writing’* no recommendations are provided.

**Box. 2 The Dutch Pharmacogenetics Working Group**

The Dutch Pharmacogenetics Working Group (DPWG) was established in 2005 by the Royal Dutch Pharmacists Association (KNMP), with the aim to develop pharmacogenetic prescribing guidelines based on systematic literature reviews and support clinicians and pharmacists in the use of genomic biomarkers for the optimisation of drug therapies. Like in the CPIC guidelines, the DPWG provides in each guidelines a grading for the quality of the evidence for the gene-drug interaction. The DPWG grading is based on a 5-point scale that ranges between 0, the lowest level of evidence, to 4, the highest level of evidence, referred to ‘*published controlled studies of good quality relating to phenotyped and/or genotyped patients or healthy volunteers, and having relevant pharmacokinetic or clinical endpoints*’ [1]. The DPWG provides also a score for the clinical relevance of the potential adverse drug event, decreased therapeutic response, or other clinical effects resulting from the gene-drug interaction. The clinical relevance is scored on a seven-point scale, derived from the National Cancer Institute’s Common Toxicity Criteria. These scale ranges from the lowest impact level of AA, assigned to clinical or pharmacokinetic effects that are not statistically significant, to the highest impact level of F, assigned to relevant clinical effects such as death, arrhythmia, or unexpected bone marrow depression [1]. Recently, the DPWG guidelines have included a clinical implication score (CIS) for specific pharmacogenetic testing, thus providing indications on when a test is deemed necessary. The CIS is based on 4 criteria, namely ‘*the clinical effect associated with gene–drug interaction; the level of evidence supporting the associated clinical effect; the number needed to genotype (NNG) in the Dutch population; the availability of and type of pharmacogenetic information in drug label issued the European Medicines Agency (EMA)’* [2]. For each criterion, a score ranging from + to +++ is assigned, to a maximum of 10+. For a total score of 3-5+, the pharmacogenetic test is regarded as beneficial whereas for total scores equal or higher than 6+ the test is deemed essential before starting a patient on a specific drug [2]. For the DPWG pharmacogenetic guidelines related to clopidogrel, we referred to the last release available through the ClinPGx database [3].

**Table S1. Inclusion of clopidogrel pharmacogenetics in the ESC guidelines and position statements published since 2011.**

| **N.** | **Guidelines and position statements** | **Type** | **Does it cite CPIC guidelines?** | **Does it cite DPWG guidelines?** | **Does it mention clopidogrel pharmacogenetics?** | **DOI** | **Ref. N.** |
| --- | --- | --- | --- | --- | --- | --- | --- |
| 1 | ESC Guidelines for the management of acute myocardial infarction in patients presenting with ST-segment elevation (2012) | GL | No | No | No | doi: 10.1093/eurheartj/ehs215 | [4] |
| 2 | 2014 ESC/EACTS Guidelines on myocardial revascularization | GL | No | No | Yes | doi: 10.1093/eurheartj/ehu278 | [5] |
| 3 | 2015 ESC Guidelines for the management of acute coronary syndromes in patients presenting without persistent ST-segment elevation | GL | No | No | No | doi: 10.1093/eurheartj/ehv320. | [6] |
| 4 | 2017 ESC focused update on dual antiplatelet therapy in coronary artery disease developed in collaboration with EACTS | PS | No | No | Yes | doi: 10.1093/eurheartj/ehx419 | [7] |
| 5 | 2017 ESC Guidelines for the management of acute myocardial infarction in patients presenting with ST-segment elevation | GL | No | No | No | doi: 10.1093/eurheartj/ehx393 | [8] |
| 6 | 2020 ESC Guidelines for the management of acute coronary syndromes in patients presenting without persistent ST-segment elevation | GL | No | No | Yes | doi: 10.1093/eurheartj/ehaa575 | [9] |
| 7 | 2023 ESC Guidelines for the management of acute coronary syndromes | GL | No | No | Yes | doi: 10.1093/eurheartj/ehad191 | [10] |
| 8 | 2024 ESC Guidelines for the management of chronic coronary syndromes | GL | No | No | Yes | doi: 10.1093/eurheartj/ehae177 | [11] |

***Abbreviations:*** GL, guidelines; PS, position statements

**Table S2. Inclusion of clopidogrel pharmacogenetics in the ACC/AHA guidelines and position statements published since 2011.**

| **N.** | **Guidelines and Position Statements** | **Type** | **Does it cite CPIC guidelines?** | **Does it cite DPWG guidelines** | **Does it mention clopidogrel pharmacogenetics?** | **DOI** | **Ref.** |
| --- | --- | --- | --- | --- | --- | --- | --- |
| 1 | 2013 ACCF/AHA Guideline for the Management of ST-Elevation Myocardial Infarction | GL | No | No | Yes | doi: 10.1161/CIR.0b013e3182742cf6 | [12] |
| 2 | 2014 AHA/ACC Guideline for the Management of Patients with Non–ST-Elevation Acute Coronary Syndromes | GL | No | No | Yes | doi: 10.1016/j.jacc.2014.09.017 | [13] |
| 3 | 2015 ACC/AHA/SCAI Focused Update on Primary Percutaneous Coronary Intervention for Patients With ST-Elevation Myocardial Infarction | GL | No | No | No | doi: 10.1161/CIR.0000000000000336 | [14] |
| 4 | 2016 ACC/AHA Guideline Focused Update on Duration of Dual Antiplatelet Therapy in Patients with Coronary Artery Disease | GL | No | No | No^a^ | doi: 10.1161/CIR.0000000000000404 | [15] |
| 5 | Updated Expert Consensus Statement on Platelet Function and Genetic Testing for Guiding P2Y12 Receptor Inhibitor Treatment in Percutaneous Coronary Intervention (2019) | PS | Yes | No | Yes | doi: 10.1016/j.jcin.2019.03.034 | [16] |
| 6 | 2021 ACC/AHA/SCAI Guideline for Coronary Artery Revascularization | GL | No | No | No | doi: 10.1016/j.jacc.2021.09.006. | [17] |
| 7 | 2023AHA/ACC/ACCP/ASPC/NLA/PCNA Guideline for the Management of Patients with Chronic Coronary Disease | GL | No | No | No | doi: 10.1161/CIR.0000000000001168 | [18] |
| 8 | Defining Strategies of Modulation of Antiplatelet Therapy in Patients with Coronary Artery Disease: A Consensus Document from the Academic Research Consortium Circulation (2023) | PS | No | No | Yes | doi: 10.1161/CIRCULATIONAHA.123.064473 | [19] |
| 9 | CYP2C19 Genetic Testing for Oral P2Y12 Inhibitor Therapy: A Scientific Statement From the American Heart Association (2024) | PS | Yes | No | Yes | DOI: 10.1161/CIR.0000000000001257 | [20] |
| 10 | International Consensus Statement on  Platelet Function and Genetic Testing in Percutaneous Coronary Intervention  2024 Update | PS | Yes | No | Yes | DOI: 10.1016/j.jcin.2024.08.027 | [21] |
| 11 | 2025 ACC/AHA/ACEP/NAEMSP/SCAI Guideline for the Management of Patients With Acute Coronary Syndromes | GL | No | No | No | doi: 10.1161/CIR.0000000000001309. | [22] |

**Notes:**

**^a^,** This guideline includes a section on the value of pharmacogenetic testing, namely section 3.4. Platelet Function Testing, Genetic Testing, and Switching of P2Y_12_ Inhibitors. No direct mention to clopidogrel is included in this section. Text from this section was included in the textual analysis, as reported in Supplementary Table S4

***Abbreviations*:** GL, guidelines; PS, position statement.

**Table S3. Textual analysis carried out on the ESC guidelines and position statements that mentioned clopidogrel pharmacogenetics. In column 1, the same number used in Supplementary Table S1 is included.**

| **N.** | **Guidelines and position statements** | **Excerpt from the guideline on clopidogrel pharmacogenetics** |
| --- | --- | --- |
| 2 | 2014 ESC/EACTS Guidelines on myocardial revascularization | **Routine platelet function testing or genetic testing** (clopidogrel and ASA) to adjust antiplatelet therapy before or after elective stenting **is not recommended** (Class III A).  Platelet function testing or genetic testing **may be considered in specific high-risk situations** (e.g. history of stent thrombosis; compliance issue; suspicion of resistance; high bleeding risk). (Class IIb C) |
| 4 | 2017 ESC focused update on dual antiplatelet  therapy in coronary artery disease developed  in collaboration with EACTS | The influence of genetic variants on the response to antiplatelet agents, especially clopidogrel, has been well-established in patients with ACS and planned PCI.  Rapidly-obtained genetic information on the (CYP)2C19 genotype can help in reaching the optimal window of P2Y12 inhibition according to the cytochrome P450 (CYP)2C19 profile, but no randomized trial has ever demonstrated any clinical benefit of such an approach Moreover, only 6–12% of the variability in on-clopidogrel platelet reactivity can be explained by the differences in genotype. For these reasons, **neither platelet function testing nor genetic testing can be recommended** for tailoring DAPT. |
| 6 | 2020 ESC Guidelines for the management of  acute coronary syndromes in patients  presenting without persistent ST-segment  elevation | De-escalation of P2Y12 receptor inhibitor treatment (e.g. with a switch from prasugrel or ticagrelor to clopidogrel) may be considered as an alternative DAPT strategy, especially for ACS patients deemed unsuitable for potent platelet inhibition. **De-escalation may be done unguided** based on clinical judgment **or guided by platelet function testing or CYP2C19 genotyping**, depending on **patient’s risk profile** and **availability of respective assays** (Class IIb A) |
| 7 | 2023 ESC Guidelines for the management  of acute coronary syndromes | In the Cost-effectiveness of CYP2C19 Genotype Guided Treatment With Antiplatelet Drugs in Patients With ST-segment-elevation Myocardial Infarction Undergoing Immediate PCI With Stent Implantation: Optimization of Treatment (POPular Genetics) trial, DAPT de-escalation from ticagrelor/prasugrel to clopidogrel guided by CYP2C19 genotyping in ACS patients undergoing PPCI within the previous 48 h was non-inferior to standard treatment with ticagrelor or prasugrel at 12 months with respect to thrombotic events and resulted in a lower incidence of bleeding.  A strategy based on **platelet function testing or genetic testing should be prospectively tested in patients who may benefit from de-escalating antithrombotic therapy.**  De-escalation of P2Y12 receptor inhibitor treatment (e.g. with a switch from prasugrel/ticagrelor to clopidogrel) may be considered as an alternative DAPT strategy to reduce bleeding risk (Class IIb A) |
| 8 | 2024 ESC Guidelines for the management  of chronic coronary syndromes | In ST-segment elevation myocardial infarction (STEMI) patients, early de-escalation from aspirin plus ticagrelor or aspirin plus prasugrel to aspirin plus clopidogrel based on genotyping or platelet function testing was non-inferior for net adverse clinical events (ischaemic endpoints and bleeding combined) compared with routine treatment with ticagrelor or prasugrel. **In patients with CCS, current evidence does not support the routine use of genotype or platelet function testing**. However, **in patients undergoing high-risk PCI who are known carriers of a *CYP2C19* loss-of-function allele**, **replacing aspirin plus clopidogrel with aspirin plus ticagrelor or prasugrel is a reasonable option.**  In **CCS patients undergoing high-thrombotic risk stenting** (e.g. complex left main stem, 2-stent bifurcation, suboptimal stenting result, prior stent thrombosis, **previously known CYP2C19 *2/*3 polymorphisms**), **prasugrel or ticagrelor** (in addition to aspirin) **may be considered instead of clopidogrel**, for the first month, and up to 3–6 months (Class IIb C) |

**Table S4. Textual analysis carried out on the ACC/AHA guidelines and position statements that mentioned clopidogrel pharmacogenetics. In column 1, the same number used in Supplementary Table S2 is included.**

| **N.** | **Guidelines and position statements** | **Excerpt from the guideline on clopidogrel pharmacogenetics** |
| --- | --- | --- |
| 1 | 2013 ACCF/AHA Guideline for the Management of STEMI | Patients who were carriers of the reduced-function CYP2C19*2 allele had significantly lower levels of the active metabolite of clopidogrel, diminished platelet inhibition, and increased rates of major adverse cardiovascular events and stent thrombosis. Other studies have not confirmed associations between CYP2C19 polymorphisms and adverse outcomes in clopidogrel-treated patients. **Future studies are needed to further clarify the risk associated with these genetic polymorphisms and to develop effective therapeutic strategies** for carriers of allelic variants of responsible enzyme systems. |
| 2 | 2014 AHA/ACC Guideline for  the Management of Patients  With Non–ST-Elevation Acute  Coronary Syndromes | A strategy of **routine genetic phenotype testing** has also not been beneficial and thus **is not recommended** |
| 4 | 2016 ACC/AHA Guideline Focused Update on Duration of Dual Antiplatelet Therapy in Patients With Coronary Artery Disease | To date, no RCT has demonstrated that routine platelet function testing or genetic testing to guide P2Y12 inhibitor therapy improves outcome; thus, **the routine use of platelet function and genetic testing is not recommended** (Class III: No Benefit) |
| 5 | Updated Expert Consensus Statement on Platelet Function and Genetic Testing for Guiding P2Y12 Receptor Inhibitor Treatment in Percutaneous Coronary Intervention (2019) | **The robustness of the evidence**, particularly when considering adequately powered randomized trials, **still does not allow recommending the use of PFT or genetic testing routinely in clinical practice**.  PFT and genetic testing may be considered as **optional tools** for guidance of treatment when **DAPT escalation or de-escalation** is required. |
| 8 | Defining Strategies of Modulation of Antiplatelet Therapy in Patients With Coronary Artery Disease: A Consensus Document from the Academic Research Consortium Circulation (2023) | **P2Y12-specific DAPT escalation (or de-escalation)** by switching **maybe guided** by clinical judgment or **by the results of platelet function testing or genotyping**.  **More data are needed on the comparative effectiveness and safety of guided versus unguided P2Y12** specific escalation (or de-escalation) by switching and the optimal timing of P2Y12-specific escalation by switching. |
| 9 | CYP2C19 Genetic Testing for Oral P2Y12 Inhibitor Therapy: A Scientific Statement From the American Heart Association (2024) | Given the totality of pharmacokinetic, pharmacodynamic, and recent clinical trial data with recent meta-analyses findings, **CYP2C19 genetic testing before prescription of clopidogrel or ticagrelor/prasugrel in patients with ACS or PCI can be beneficial**. The implementation of CYP2C19 genetic testing for individualizing oral P2Y12 inhibitor therapy depends on clinician and patient perceptions, recommendations provided by clinical guidelines that incorporate recently published clinical evidence. |
| 10 | International Consensus Statement on Platelet Function and Genetic Testing in Percutaneous Coronary Intervention: 2024 Update | The **robustness of evidence** on guided escalation or de-escalation of P2Y12 inhibiting therapy **still does not allow recommending the use of PFT or GT as the preferred strategy for all PCI patients.** Nevertheless, there is still sufficient evidence to consider their use in clinical practice, particularly in **specific clinical settings**, taking into account also the pretest probability of adverse events of the individual patient (ie, **bleeding and ischemic risks**), with an approach (i.e., escalation vs de-escalation) chosen according to the clinical presentation (CCS vs ACS). |

**Table S5. Analysis of the areas of expertise and involvement in clopidogrel pharmacogenomics amongst the authors of the ESC guidelines.**

| **N.** | **Guidelines and Position Statements** | **N. of authors** | **N. of Geneticists / Pharmacologist** | **Mention of Clopidogrel PGx in their publications** | **Ref. N.** |
| --- | --- | --- | --- | --- | --- |
| 1 | ESC Guidelines for the management of acute myocardial infarction in patients presenting with ST-segment elevation (2012) | 23 | 0 | No | [4] |
| 2 | 2014 ESC/EACTS Guidelines on myocardial revascularization | 25 | 0 | Yes | [5] |
| 3 | 2015 ESC Guidelines for the management of acute coronary syndromes in patients presenting without persistent ST-segment elevation | 19 | 1 | Yes | [6] |
| 4 | 2017 ESC focused update on dual antiplatelet therapy in coronary artery disease developed in collaboration with EACTS | 18 | 0 | Yes | [7] |
| 5 | 2017 ESC Guidelines for the management of acute myocardial infarction in patients presenting with ST-segment elevation | 19 | 0 | No | [8] |
| 6 | 2020 ESC Guidelines for the management of acute coronary syndromes in patients presenting without persistent ST-segment elevation | 24 | 0 | Yes | [9] |
| 7 | 2023 ESC Guidelines for the management of acute coronary syndromes | 26 | 0 | Yes | [10] |
| 8 | 2024 ESC Guidelines for the management of chronic coronary syndromes | 28 | 0 | Yes | [11] |

**Table S6. Analysis of the areas of expertise and involvement in clopidogrel pharmacogenomics amongst the authors of the ACC/AHA guidelines**

| **N.** | **Guidelines and Position Statements** | **N. of Authors** | **N. of Geneticists / Pharmacologists** | **Mention of Clopidogrel PGx in their publications** | **Ref. N.** |
| --- | --- | --- | --- | --- | --- |
| 1 | 2013 ACCF/AHA Guideline for the Management of ST-Elevation Myocardial Infarction | 23 | 1 | No | [12] |
| 2 | 2014 AHA/ACC Guideline for the Management of Patients with Non–ST-Elevation Acute Coronary Syndromes | 17 | 0 | Yes | [13] |
| 3 | 2015 ACC/AHA/SCAI Focused Update on Primary Percutaneous Coronary Intervention for Patients With ST-Elevation Myocardial Infarction | 40 | 0 | No | [14] |
| 4 | 2016 ACC/AHA Guideline Focused Update on Duration of Dual Antiplatelet Therapy in Patients with Coronary Artery Disease | 17 | 0 | No | [15] |
| 5 | Updated Expert Consensus Statement on Platelet Function and Genetic Testing for Guiding P2Y12 Receptor Inhibitor Treatment in Percutaneous Coronary Intervention (2019) | 26 |  | Yes | [16] |
| 6 | 2021 ACC/AHA/SCAI Guideline for Coronary Artery Revascularization | 24 | 0 | No | [17] |
| 7 | 2023AHA/ACC/ACCP/ASPC/NLA/PCNA Guideline for the Management of Patients with Chronic Coronary NoDisease | 27 | 0 | Yes | [18] |
| 8 | Defining Strategies of Modulation of Antiplatelet Therapy in Patients with Coronary Artery Disease: A Consensus Document from the Academic Research Consortium Circulation (2023) | 41 | 0 | Yes | [19] |
| 9 | CYP2C19 Genetic Testing for Oral P2Y12 Inhibitor Therapy: A Scientific Statement From the American Heart Association (2024) | 12 | 4 | Yes | [20] |
| 10 | International Consensus Statement on Platelet Function and Genetic Testing in Percutaneous Coronary Intervention: 2024 Update | 33 | 1 | Yes | [21] |
| 11 | 2025 ACC/AHA/ACEP/NAEMSP/SCAI Guideline for the Management of Patients With Acute Coronary Syndromes | 30 | 1 | No | [22] |

**Supplementary References**

1. Swen JJ, Wilting I, de Goede AL et al. Pharmacogenetics: from bench to byte. *Clin Pharmacol Ther*. 2008; 83: 781-787. doi: 10.1038/sj.clpt.6100507.
2. Swen JJ, Nijenhuis M, van Rhenen M et al. Pharmacogenetic Information in Clinical Guidelines: The European Perspective. *Clin Pharmacol Ther*. 2018; 103: 795-801. doi: 10.1002/cpt.1049.
3. The ClinPGx database - <https://www.clinpgx.org/> (Accessed on 31 March 2026).
4. Task Force on the management of ST-segment elevation acute myocardial infarction of the European Society of Cardiology (ESC); Steg PG, James SK, Atar D, Badano LP, Blömstrom-Lundqvist C, Borger MA, Di Mario C, Dickstein K, Ducrocq G, Fernandez-Aviles F, Gershlick AH, Giannuzzi P, Halvorsen S, Huber K, Juni P, Kastrati A, Knuuti J, Lenzen MJ, Mahaffey KW, Valgimigli M, van 't Hof A, Widimsky P, Zahger D. ESC Guidelines for the management of acute myocardial infarction in patients presenting with ST-segment elevation. *Eur Heart J*. 2012; 33: 2569-2619. doi: 10.1093/eurheartj/ehs215.
5. Authors/Task Force members; Windecker S, Kolh P, Alfonso F, Collet JP, Cremer J, Falk V, Filippatos G, Hamm C, Head SJ, Jüni P, Kappetein AP, Kastrati A, Knuuti J, Landmesser U, Laufer G, Neumann FJ, Richter DJ, Schauerte P, Sousa Uva M, Stefanini GG, Taggart DP, Torracca L, Valgimigli M, Wijns W, Witkowski A. 2014 ESC/EACTS Guidelines on myocardial revascularization: The Task Force on Myocardial Revascularization of the European Society of Cardiology (ESC) and the European Association for Cardio-Thoracic Surgery (EACTS) Developed with the special contribution of the European Association of Percutaneous Cardiovascular Interventions (EAPCI). *Eur Heart J*. 2014; 35: 2541-2619. doi: 10.1093/eurheartj/ehu278.
6. Roffi M, Patrono C, Collet JP, Mueller C, Valgimigli M, Andreotti F, Bax JJ, Borger MA, Brotons C, Chew DP, Gencer B, Hasenfuss G, Kjeldsen K, Lancellotti P, Landmesser U, Mehilli J, Mukherjee D, Storey RF, Windecker S; ESC Scientific Document Group. 2015 ESC Guidelines for the management of acute coronary syndromes in patients presenting without persistent ST-segment elevation: Task Force for the Management of Acute Coronary Syndromes in Patients Presenting without Persistent ST-Segment Elevation of the European Society of Cardiology (ESC). *Eur Heart J*. 2016; 37: 267-315. doi: 10.1093/eurheartj/ehv320.
7. Valgimigli M, Bueno H, Byrne RA, Collet JP, Costa F, Jeppsson A, Jüni P, Kastrati A, Kolh P, Mauri L, Montalescot G, Neumann FJ, Petricevic M, Roffi M, Steg PG, Windecker S, Zamorano JL, Levine GN; ESC Scientific Document Group; ESC Committee for Practice Guidelines (CPG); ESC National Cardiac Societies. 2017 ESC focused update on dual antiplatelet therapy in coronary artery disease developed in collaboration with EACTS: The Task Force for dual antiplatelet therapy in coronary artery disease of the European Society of Cardiology (ESC) and of the European Association for Cardio-Thoracic Surgery (EACTS). *Eur Heart J*. 2018; 39:213-260. doi: 10.1093/eurheartj/ehx419.
8. Ibanez B, James S, Agewall S, Antunes MJ, Bucciarelli-Ducci C, Bueno H, Caforio ALP, Crea F, Goudevenos JA, Halvorsen S, Hindricks G, Kastrati A, Lenzen MJ, Prescott E, Roffi M, Valgimigli M, Varenhorst C, Vranckx P, Widimský P; ESC Scientific Document Group. 2017 ESC Guidelines for the management of acute myocardial infarction in patients presenting with ST-segment elevation: The Task Force for the management of acute myocardial infarction in patients presenting with ST-segment elevation of the European Society of Cardiology (ESC). *Eur Heart J*. 2018; 39:119-177. doi: 10.1093/eurheartj/ehx393.
9. Collet JP, Thiele H, Barbato E, Barthélémy O, Bauersachs J, Bhatt DL, Dendale P, Dorobantu M, Edvardsen T, Folliguet T, Gale CP, Gilard M, Jobs A, Jüni P, Lambrinou E, Lewis BS, Mehilli J, Meliga E, Merkely B, Mueller C, Roffi M, Rutten FH, Sibbing D, Siontis GCM; ESC Scientific Document Group. 2020 ESC Guidelines for the management of acute coronary syndromes in patients presenting without persistent ST-segment elevation. *Eur Heart J*. 2021; 42:1289-1367. doi: 10.1093/eurheartj/ehaa575.
10. Byrne RA, Rossello X, Coughlan JJ, Barbato E, Berry C, Chieffo A, Claeys MJ, Dan GA, Dweck MR, Galbraith M, Gilard M, Hinterbuchner L, Jankowska EA, Jüni P, Kimura T, Kunadian V, Leosdottir M, Lorusso R, Pedretti RFE, Rigopoulos AG, Rubini Gimenez M, Thiele H, Vranckx P, Wassmann S, Wenger NK, Ibanez B; ESC Scientific Document Group. 2023 ESC Guidelines for the management of acute coronary syndromes. *Eur Heart J*. 2023;44:3720-3826. doi: 10.1093/eurheartj/ehad191.
11. Vrints C, Andreotti F, Koskinas KC, Rossello X, Adamo M, Ainslie J, Banning AP, Budaj A, Buechel RR, Chiariello GA, Chieffo A, Christodorescu RM, Deaton C, Doenst T, Jones HW, Kunadian V, Mehilli J, Milojevic M, Piek JJ, Pugliese F, Rubboli A, Semb AG, Senior R, Ten Berg JM, Van Belle E, Van Craenenbroeck EM, Vidal-Perez R, Winther S; ESC Scientific Document Group. 2024 ESC Guidelines for the management of chronic coronary syndromes. *Eur Heart J*. 2024; 45: 3415-3537. doi: 10.1093/eurheartj/ehae177.
12. O'Gara PT, Kushner FG, Ascheim DD, Casey DE Jr, Chung MK, de Lemos JA, Ettinger SM, Fang JC, Fesmire FM, Franklin BA, Granger CB, Krumholz HM, Linderbaum JA, Morrow DA, Newby LK, Ornato JP, Ou N, Radford MJ, Tamis-Holland JE, Tommaso CL, Tracy CM, Woo YJ, Zhao DX, Anderson JL, Jacobs AK, Halperin JL, Albert NM, Brindis RG, Creager MA, DeMets D, Guyton RA, Hochman JS, Kovacs RJ, Kushner FG, Ohman EM, Stevenson WG, Yancy CW; American College of Cardiology Foundation/American Heart Association Task Force on Practice Guidelines. 2013 ACCF/AHA guideline for the management of ST-elevation myocardial infarction: a report of the American College of Cardiology Foundation/American Heart Association Task Force on Practice Guidelines. *Circulation*. 2013;127:e362-425. doi: 10.1161/CIR.0b013e3182742cf6.
13. Amsterdam EA, Wenger NK, Brindis RG, Casey DE Jr, Ganiats TG, Holmes DR Jr, Jaffe AS, Jneid H, Kelly RF, Kontos MC, Levine GN, Liebson PR, Mukherjee D, Peterson ED, Sabatine MS, Smalling RW, Zieman SJ. 2014 AHA/ACC Guideline for the Management of Patients with Non-ST-Elevation Acute Coronary Syndromes: a report of the American College of Cardiology/American Heart Association Task Force on Practice Guidelines. *J Am Coll Cardiol*. 2014;64:e139-e228. doi: 10.1016/j.jacc.2014.09.017.
14. Levine GN, Bates ER, Blankenship JC, Bailey SR, Bittl JA, Cercek B, Chambers CE, Ellis SG, Guyton RA, Hollenberg SM, Khot UN, Lange RA, Mauri L, Mehran R, Moussa ID, Mukherjee D, Ting HH, O'Gara PT, Kushner FG, Ascheim DD, Brindis RG, Casey DE Jr, Chung MK, de Lemos JA, Diercks DB, Fang JC, Franklin BA, Granger CB, Krumholz HM, Linderbaum JA, Morrow DA, Newby LK, Ornato JP, Ou N, Radford MJ, Tamis-Holland JE, Tommaso CL, Tracy CM, Woo YJ, Zhao DX. 2015 ACC/AHA/SCAI Focused Update on Primary Percutaneous Coronary Intervention for Patients With ST-Elevation Myocardial Infarction: An Update of the 2011 ACCF/AHA/SCAI Guideline for Percutaneous Coronary Intervention and the 2013 ACCF/AHA Guideline for the Management of ST-Elevation Myocardial Infarction: A Report of the American College of Cardiology/American Heart Association Task Force on Clinical Practice Guidelines and the Society for Cardiovascular Angiography and Interventions. *Circulation*. 2016; 133:1135-1147. doi: 10.1161/CIR.0000000000000336.
15. Levine GN, Bates ER, Bittl JA, Brindis RG, Fihn SD, Fleisher LA, Granger CB, Lange RA, Mack MJ, Mauri L, Mehran R, Mukherjee D, Newby LK, O'Gara PT, Sabatine MS, Smith PK, Smith SC Jr. 2016 ACC/AHA Guideline Focused Update on Duration of Dual Antiplatelet Therapy in Patients With Coronary Artery Disease: A Report of the American College of Cardiology/American Heart Association Task Force on Clinical Practice Guidelines: An Update of the 2011 ACCF/AHA/SCAI Guideline for Percutaneous Coronary Intervention, 2011 ACCF/AHA Guideline for Coronary Artery Bypass Graft Surgery, 2012 ACC/AHA/ACP/AATS/PCNA/SCAI/STS Guideline for the Diagnosis and Management of Patients With Stable Ischemic Heart Disease, 2013 ACCF/AHA Guideline for the Management of ST-Elevation Myocardial Infarction, 2014 AHA/ACC Guideline for the Management of Patients With Non-ST-Elevation Acute Coronary Syndromes, and 2014 ACC/AHA Guideline on Perioperative Cardiovascular Evaluation and Management of Patients Undergoing Noncardiac Surgery. *Circulation*. 2016; 134:e123-55. doi: 10.1161/CIR.0000000000000404.
16. Sibbing D, Aradi D, Alexopoulos D, Ten Berg J, Bhatt DL, Bonello L, Collet JP, Cuisset T, Franchi F, Gross L, Gurbel P, Jeong YH, Mehran R, Moliterno DJ, Neumann FJ, Pereira NL, Price MJ, Sabatine MS, So DYF, Stone GW, Storey RF, Tantry U, Trenk D, Valgimigli M, Waksman R, Angiolillo DJ. Updated Expert Consensus Statement on Platelet Function and Genetic Testing for Guiding P2Y_12_ Receptor Inhibitor Treatment in Percutaneous Coronary Intervention. *JACC Cardiovasc Interv*. 2019;12: 1521-1537. doi: 10.1016/j.jcin.2019.03.034.
17. Writing Committee Members; Lawton JS, Tamis-Holland JE, Bangalore S, Bates ER, Beckie TM, Bischoff JM, Bittl JA, Cohen MG, DiMaio JM, Don CW, Fremes SE, Gaudino MF, Goldberger ZD, Grant MC, Jaswal JB, Kurlansky PA, Mehran R, Metkus TS Jr, Nnacheta LC, Rao SV, Sellke FW, Sharma G, Yong CM, Zwischenberger BA. 2021 ACC/AHA/SCAI Guideline for Coronary Artery Revascularization: A Report of the American College of Cardiology/American Heart Association Joint Committee on Clinical Practice Guidelines. *J Am Coll Cardiol*. 2022; 79: e21-e129. doi: 10.1016/j.jacc.2021.09.006.
18. Virani SS, Newby LK, Arnold SV, Bittner V, Brewer LC, Demeter SH, Dixon DL, Fearon WF, Hess B, Johnson HM, Kazi DS, Kolte D, Kumbhani DJ, LoFaso J, Mahtta D, Mark DB, Minissian M, Navar AM, Patel AR, Piano MR, Rodriguez F, Talbot AW, Taqueti VR, Thomas RJ, van Diepen S, Wiggins B, Williams MS; Peer Review Committee Members. 2023 AHA/ACC/ACCP/ASPC/NLA/PCNA Guideline for the Management of Patients With Chronic Coronary Disease: A Report of the American Heart Association/American College of Cardiology Joint Committee on Clinical Practice Guidelines. *Circulation*. 2023; 148: e9-e119. doi: 10.1161/CIR.0000000000001168.
19. Capodanno D, Mehran R, Krucoff MW, Baber U, Bhatt DL, Capranzano P, Collet JP, Cuisset T, De Luca G, De Luca L, Farb A, Franchi F, Gibson CM, Hahn JY, Hong MK, James S, Kastrati A, Kimura T, Lemos PA, Lopes RD, Magee A, Matsumura R, Mochizuki S, O'Donoghue ML, Pereira NL, Rao SV, Rollini F, Shirai Y, Sibbing D, Smits PC, Steg PG, Storey RF, Ten Berg J, Valgimigli M, Vranckx P, Watanabe H, Windecker S, Serruys PW, Yeh RW, Morice MC, Angiolillo DJ. Defining Strategies of Modulation of Antiplatelet Therapy in Patients With Coronary Artery Disease: A Consensus Document from the Academic Research Consortium. *Circulation*. 2023; 147: 1933-1944. doi: 10.1161/CIRCULATIONAHA.123.064473.
20. Pereira NL, Cresci S, Angiolillo DJ, Batchelor W, Capers Q 4th, Cavallari LH, Leifer D, Luzum JA, Roden DM, Stellos K, Turrise SL, Tuteja S; American Heart Association Professional/Public Education and Publications Committee of the Council on Genomic and Precision Medicine; Council on Arteriosclerosis, Thrombosis and Vascular Biology; Council on Cardiovascular and Stroke Nursing; Council on Clinical Cardiology; Council on Peripheral Vascular Disease; and Stroke Council. *CYP2C19* Genetic Testing for Oral P2Y12 Inhibitor Therapy: A Scientific Statement From the American Heart Association. *Circulation*. 2024;150: e129-e150. doi: 10.1161/CIR.0000000000001257.
21. Angiolillo DJ, Galli M, Alexopoulos D, Aradi D, Bhatt DL, Bonello L, Capodanno D, Cavallari LH, Collet JP, Cuisset T, Ferreiro JL, Franchi F, Geisler T, Gibson CM, Gorog DA, Gurbel PA, Jeong YH, Marcucci R, Siller-Matula JM, Mehran R, Neumann FJ, Pereira NL, Rizas KD, Rollini F, So DYF, Stone GW, Storey RF, Tantry US, Berg JT, Trenk D, Valgimigli M, Waksman R, Sibbing D. International Consensus Statement on Platelet Function and Genetic Testing in Percutaneous Coronary Intervention: 2024 Update. *JACC Cardiovasc Interv*. 2024;17: 2639-2663. doi: 10.1016/j.jcin.2024.08.027.
22. Rao SV, O'Donoghue ML, Ruel M, Rab T, Tamis-Holland JE, Alexander JH, Baber U, Baker H, Cohen MG, Cruz-Ruiz M, Davis LL, de Lemos JA, DeWald TA, Elgendy IY, Feldman DN, Goyal A, Isiadinso I, Menon V, Morrow DA, Mukherjee D, Platz E, Promes SB, Sandner S, Sandoval Y, Schunder R, Shah B, Stopyra JP, Talbot AW, Taub PR, Williams MS. 2025 ACC/AHA/ACEP/NAEMSP/SCAI Guideline for the Management of Patients With Acute Coronary Syndromes: A Report of the American College of Cardiology/American Heart Association Joint Committee on Clinical Practice Guidelines. *Circulation*. 2025;151: e771-e862. doi: 10.1161/CIR.0000000000001309.
