# Supplementary material for: Limited Visibility and Perception of the Clinical Relevance of Clopidogrel Pharmacogenetics in Cardiology Literature
Source: Clin Transl Sci. 2026 May 11;19(5):e70584. doi: 10.1111/cts.70584 (PMC13160923; doi:10.1111/cts.70584)
Supplement: Supplementary file 2 — Data S1: cts70584‐sup‐0002‐DataS1.pdf. [file CTS-19-e70584-s003.pdf]

Citations - 2022 CPIC Guidelines			
PMID	Citation	First Author	Publication Year
38003001	Genes (Basel). 2023 Nov 9;14(11):2057. doi: 10.3390/genes14112057.	Mauriello A	2023
36154845	Hum Genomics. 2022 Sep 25;16(1):42. doi: 10.1186/s40246-022-00417-9.	Al-Mahayri ZN	2022
36685861	Front Genet. 2023 Jan 4;13:1070236. doi: 10.3389/fgene.2022.1070236. eCollection 2022.	Sun B	2023
36817149	Front Pharmacol. 2023 Feb 2;14:1110460. doi: 10.3389/fphar.2023.1110460. eCollection 2023.	Calleja S	2023
35814245	Front Pharmacol. 2022 Jun 24;13:939313. doi: 10.3389/fphar.2022.939313. eCollection 2022.	Blazy C	2022
37895189	Genes (Basel). 2023 Sep 22;14(10):1841. doi: 10.3390/genes14101841.	van der Drift D	2023
37929326	Pharmacogenomics. 2023 Nov;24(16):845-857. doi: 10.2217/pgs-2023-0166. Epub 2023 Nov 6.	Booyse RP	2023
38765788	Pharmgenomics Pers Med. 2024 May 14;17:225-236. doi: 10.2147/PGPM.S457805. eCollection 2024.	Teng JM	2024
36980851	Genes (Basel). 2023 Feb 25;14(3):578. doi: 10.3390/genes14030578.	Levens AD	2023
39544755	Am J Transl Res. 2024 Oct 15;16(10):5708-5717. doi: 10.62347/EWUH3396. eCollection 2024.	Chen S	2024
39576732	Clin Transl Sci. 2024 Nov;17(11):e70079. doi: 10.1111/cts.70079.	Lewis JP	2024
38253063	Am J Health Syst Pharm. 2024 Jun 11;81(12):555-562. doi: 10.1093/ajhp/zxae008.	Aquilante CL	2024
38550953	Camb Prism Precis Med. 2023 Jun 29;1:e28. doi: 10.1017/pcm.2023.17. eCollection 2023.	Padmanabhan S	2023
41097034	Int J Mol Sci. 2025 Oct 7;26(19):9766. doi: 10.3390/ijms26199766.	Antúnez-Rodríguez A	2025
40611487	J Am Heart Assoc. 2025 Jul 15;14(14):e041634. doi: 10.1161/JAHA.125.041634. Epub 2025 Jul 3.	Patel RC	2025
35912831	Expert Rev Clin Pharmacol. 2022 Jul;15(7):811-825. doi: 10.1080/17512433.2022.2108401. Epub 2022 Aug 4.	McDermott JH	2022
36913975	Thromb Haemost. 2023 Aug;123(8):808-839. doi: 10.1055/a-2052-9175. Epub 2023 Mar 13.	Akbulut AC	2023
35748236	Per Med. 2022 Jul;19(4):327-339. doi: 10.2217/pme-2021-0175. Epub 2022 Jun 24.	Alrajeh KY	2022
37215094	Res Pract Thromb Haemost. 2023 Mar 28;7(3):100138. doi: 10.1016/j.rpth.2023.100138. eCollection 2023 Mar.	Rocca B	2023
37284308	Front Pharmacol. 2023 May 22;14:1180640. doi: 10.3389/fphar.2023.1180640. eCollection 2023.	Samarasinghe SR	2023
39582510	J Am Coll Clin Pharm. 2024 Jun;7(6):581-588. doi: 10.1002/jac5.1958. Epub 2024 May 8.	Donnelly RS	2024
38899464	Circulation. 2024 Aug 6;150(6):e129-e150. doi: 10.1161/CIR.0000000000001257. Epub 2024 Jun 20.	Pereira NL	2024
38818903	Clin Transl Sci. 2024 Jun;17(6):e13800. doi: 10.1111/cts.13800.	Abdel-Latif R	2024
39260933	J Am Coll Cardiol. 2024 Sep 17;84(12):1107-1118. doi: 10.1016/j.jacc.2024.06.038.	van den Broek WWA	2024
38420192	Front Pharmacol. 2024 Feb 14;15:1326776. doi: 10.3389/fphar.2024.1326776. eCollection 2024.	Shubbar Q	2024
36927865	Nat Commun. 2023 Mar 17;14(1):1474. doi: 10.1038/s41467-023-37209-1.	Powell NR	2023
37547044	Lancet Reg Health West Pac. 2023 May 22;36:100779. doi: 10.1016/j.lanwpc.2023.100779. eCollection 2023 Ju	Huang Q	2023
40351093	Clin Pharmacol Ther. 2025 Aug;118(2):470-479. doi: 10.1002/cpt.3698. Epub 2025 May 12.	Springer AN	2025
40756326	Genet Med Open. 2025 Jun 30;3:103441. doi: 10.1016/j.gimo.2025.103441. eCollection 2025.	Morales A	2025

40715000	J Clin Lab Anal. 2025 Sep;39(18):e70088. doi: 10.1002/jcla.70088. Epub 2025 Jul 25.	Wei G	2025
35429163	Clin Pharmacol Ther. 2022 Jul;112(1):146-155. doi: 10.1002/cpt.2612. Epub 2022 May 2.	Thomas CD	2022
41027428	Am J Hum Genet. 2025 Nov 6;112(11):2708-2719. doi: 10.1016/j.ajhg.2025.09.006. Epub 2025 Sep 29.	Singh A	2025
39462319	BMC Geriatr. 2024 Oct 26;24(1):881. doi: 10.1186/s12877-024-05471-7.	Zhang S	2024
38003881	J Pers Med. 2023 Oct 31;13(11):1566. doi: 10.3390/jpm13111566.	Villapalos-García G	2023
38388691	NPJ Genom Med. 2024 Feb 22;9(1):13. doi: 10.1038/s41525-024-00403-1.	Kennedy A	2024
39161448	RSC Adv. 2024 Aug 19;14(36):26007-26015. doi: 10.1039/d4ra03913k. eCollection 2024 Aug 16.	Wang H	2024
38482263	Balkan J Med Genet. 2024 Mar 12;26(2):35-40. doi: 10.2478/bjmg-2023-0023. eCollection 2023 Dec.	Nestorovska KA	2024
39617913	Hum Genomics. 2024 Dec 2;18(1):135. doi: 10.1186/s40246-024-00699-1.	Li CX	2024
38038819	Cardiovasc Drugs Ther. 2024 Dec;38(6):1397-1407. doi: 10.1007/s10557-023-07534-0. Epub 2023 Dec 1.	Cargnin S	2024
39746327	Cerebrovasc Dis Extra. 2025;15(1):68-72. doi: 10.1159/000543331. Epub 2025 Jan 2.	Kittipanprayoon S	2025
40612749	Front Pharmacol. 2025 Jun 19;16:1572886. doi: 10.3389/fphar.2025.1572886. eCollection 2025.	Hardi H	2025
36506510	Front Pharmacol. 2022 Nov 24;13:1007113. doi: 10.3389/fphar.2022.1007113. eCollection 2022.	Campos-Staffico AM	2022
38599713	J Am Coll Cardiol. 2024 Apr 16;83(15):1370-1381. doi: 10.1016/j.jacc.2024.02.015.	Thomas CD	2024
38424298	Eur J Hum Genet. 2024 Aug;32(8):895-902. doi: 10.1038/s41431-024-01567-1. Epub 2024 Feb 29.	Massmann A	2024
39672782	Trends Pharmacol Sci. 2025 Jan;46(1):78-93. doi: 10.1016/j.tips.2024.11.005. Epub 2024 Dec 12.	Jain K	2025
37987380	Pharmacy (Basel). 2023 Oct 26;11(6):170. doi: 10.3390/pharmacy11060170.	Ghazal H	2023
36611001	J Appl Lab Med. 2023 Mar 6;8(2):251-263. doi: 10.1093/jalm/jfac091.	Williams GR	2023
35444556	Front Pharmacol. 2022 Apr 4;13:866058. doi: 10.3389/fphar.2022.866058. eCollection 2022.	Asiimwe IG	2022
37287029	Hum Genomics. 2023 Jun 7;17(1):51. doi: 10.1186/s40246-023-00495-3.	Koufaki MI	2023
38790236	Genes (Basel). 2024 May 10;15(5):607. doi: 10.3390/genes15050607.	Ganoci L	2024
39734623	JVS Vasc Insights. 2024;2:100112. doi: 10.1016/j.jvsvi.2024.100112.	Burke KA	2024
40330492	Pharmgenomics Pers Med. 2025 May 2;18:105-113. doi: 10.2147/PGPM.S509794. eCollection 2025.	Li XP	2025
40095636	Clin Appl Thromb Hemost. 2025 Jan-Dec;31:10760296251327594. doi: 10.1177/10760296251327594. Epub 20	Hu B	2025
40423057	J Pers Med. 2025 May 2;15(5):185. doi: 10.3390/jpm15050185.	Radwan A	2025
39443518	Nat Commun. 2024 Oct 23;15(1):9156. doi: 10.1038/s41467-024-53556-z.	Cordioli M	2024
40573293	Pharmaceuticals (Basel). 2025 Jun 16;18(6):898. doi: 10.3390/ph18060898.	Pop C	2025
39258919	Pharmacogenomics. 2024;25(8-9):391-399. doi: 10.1080/14622416.2024.2394014. Epub 2024 Sep 11.	Massmann A	2024
39245547	Interv Cardiol Clin. 2024 Oct;13(4):469-481. doi: 10.1016/j.iccl.2024.06.002. Epub 2024 Aug 1.	Cavallari LH	2024
37963685	Open Heart. 2023 Nov;10(2):e002436. doi: 10.1136/openhrt-2023-002436.	Lopez J	2023
36601065	Front Cardiovasc Med. 2022 Dec 19;9:1016126. doi: 10.3389/fcvm.2022.1016126. eCollection 2022.	Dai R	2022

37479695	Cell Discov. 2023 Jul 21;9(1):75. doi: 10.1038/s41421-023-00582-8.	Cheng S	2023
39537191	Eur Heart J Cardiovasc Pharmacother. 2025 May 2;11(3):230-240. doi: 10.1093/ehjcvp/pvae087.	van den Broek WWA	2025
39901689	Curr Cardiol Rev. 2025;21(4):e1573403X334668. doi: 10.2174/011573403X334668241227074314.	Kayani M	2025
38390792	J Am Heart Assoc. 2024 Mar 5;13(5):e030058. doi: 10.1161/JAHA.123.030058. Epub 2024 Feb 23.	Lim KK	2024
38853370	Clin Transl Sci. 2024 Jun;17(6):e13830. doi: 10.1111/cts.13830.	John S	2024
39868839	Clin Pharmacol Ther. 2025 May;117(5):1313-1324. doi: 10.1002/cpt.3552. Epub 2025 Jan 27.	Yang G	2025
41059196	Front Pharmacol. 2025 Sep 22;16:1659875. doi: 10.3389/fphar.2025.1659875. eCollection 2025.	Coumau A	2025
37954650	Int J Gen Med. 2023 Nov 6;16:5139-5146. doi: 10.2147/IJGM.S437251. eCollection 2023.	Xie J	2023
36588476	Pharmacotherapy. 2023 Feb;43(2):158-175. doi: 10.1002/phar.2758. Epub 2023 Jan 13.	Thomas CD	2023
37920809	Int J Cardiol Cardiovasc Risk Prev. 2023 Oct 19;19:200222. doi: 10.1016/j.ijcrp.2023.200222. eCollection 2023	Hoang Ngo T	2023
38555869	Ann R Coll Surg Engl. 2024 Apr;106(4):321-328. doi: 10.1308/rcsann.2024.0031.	Alkhatib O	2024
37116171	Adv Sci (Weinh). 2023 Jul;10(20):e2206343. doi: 10.1002/advs.202206343. Epub 2023 Apr 28.	Liu J	2023
37067798	JAMA Netw Open. 2023 Apr 3;6(4):e238585. doi: 10.1001/jamanetworkopen.2023.8585.	Wang Y	2023
39235670	Drugs. 2024 Oct;84(10):1275-1297. doi: 10.1007/s40265-024-02076-7. Epub 2024 Sep 5.	Maas DPMSM	2024
39702032	BMC Cardiovasc Disord. 2024 Dec 19;24(1):713. doi: 10.1186/s12872-024-04399-5.	Wang Q	2024
39584620	Clin Pharmacol Ther. 2025 Feb;117(2):387-397. doi: 10.1002/cpt.3502. Epub 2024 Nov 25.	Lebreton L	2025
39418209	QJM. 2025 Mar 1;118(3):154-160. doi: 10.1093/qjmed/hcae200.	McDermott JH	2025
40661922	Pharmgenomics Pers Med. 2025 Jul 9;18:163-177. doi: 10.2147/PGPM.S519342. eCollection 2025.	Dou N	2025
39847465	Annu Rev Pharmacol Toxicol. 2025 Jan;65(1):111-130. doi: 10.1146/annurev-pharmtox-061724-080718.	Empey PE	2025
40831648	Fed Pract. 2025 May;42(5):200-203. doi: 10.12788/fp.0554. Epub 2025 May 17.	Sens HM	2025
38173046	Hum Genomics. 2024 Jan 3;18(1):2. doi: 10.1186/s40246-023-00568-3.	Khasawneh LQ	2024
36744212	Front Pharmacol. 2023 Jan 18;13:931405. doi: 10.3389/fphar.2022.931405. eCollection 2022.	Zhang X	2023
36818341	Front Cardiovasc Med. 2023 Feb 2;10:1020593. doi: 10.3389/fcvm.2023.1020593. eCollection 2023.	Mo Y	2023
36760562	Front Cardiovasc Med. 2023 Jan 24;10:1105001. doi: 10.3389/fcvm.2023.1105001. eCollection 2023.	Cheng Y	2023
36713827	Front Pharmacol. 2023 Jan 13;14:1101240. doi: 10.3389/fphar.2023.1101240. eCollection 2023.	Shao R	2023
36778023	Front Pharmacol. 2023 Jan 26;14:1008330. doi: 10.3389/fphar.2023.1008330. eCollection 2023.	Liu Y	2023
37025454	Front Genet. 2023 Mar 21;14:1114742. doi: 10.3389/fgene.2023.1114742. eCollection 2023.	Fernandes VC	2023
36976586	West J Emerg Med. 2023 Feb 25;24(2):312-321. doi: 10.5811/westjem.2022.11.57981.	Vest A	2023
38495361	Innov Pharm. 2023 Nov 20;14(4):10.24926/iip.v14i4.5796. doi: 10.24926/iip.v14i4.5796. eCollection 2023.	Olson AW	2023
38581109	Clin Transl Sci. 2024 Apr;17(4):e13792. doi: 10.1111/cts.13792.	Biswas M	2024
36077453	Int J Mol Sci. 2022 Sep 2;23(17):10058. doi: 10.3390/ijms231710058.	Idda ML	2022

40650174	Int J Mol Sci. 2025 Jul 3;26(13):6397. doi: 10.3390/ijms26136397.	Casaburi G	2025
40724525	Life (Basel). 2025 Jun 27;15(7):1023. doi: 10.3390/life15071023.	Juginović A	2025
40940774	Cells. 2025 Sep 1;14(17):1363. doi: 10.3390/cells14171363.	Rzeczycki P	2025
41028360	Sci Rep. 2025 Sep 30;15(1):33765. doi: 10.1038/s41598-025-96338-3.	Bharath G	2025
39360670	Pharmacogenomics. 2024;25(12-13):503-513. doi: 10.1080/14622416.2024.2406213. Epub 2024 Oct 3.	Fox LP	2024
36068297	Pharmacogenomics J. 2022 Dec;22(5-6):284-293. doi: 10.1038/s41397-022-00288-2. Epub 2022 Sep 6.	Zhou Y	2022
38285330	Cardiol Ther. 2024 Mar;13(1):191-203. doi: 10.1007/s40119-024-00348-7. Epub 2024 Jan 29.	Jones D	2024
36297437	Pharmaceutics. 2022 Sep 21;14(10):2001. doi: 10.3390/pharmaceutics14102001.	Zubiaur P	2022
36905169	IET Nanobiotechnol. 2023 May;17(3):281-288. doi: 10.1049/nbt2.12123. Epub 2023 Mar 10.	Li Y	2023
35373152	HGG Adv. 2022 Mar 16;3(2):100100. doi: 10.1016/j.xhgg.2022.100100. eCollection 2022 Apr 14.	Auwerx C	2022
37775990	CPT Pharmacometrics Syst Pharmacol. 2024 Jan;13(1):29-40. doi: 10.1002/psp4.13053. Epub 2023 Oct 4.	Jung YS	2024
38645949	Radiol Case Rep. 2024 Apr 13;19(7):2629-2632. doi: 10.1016/j.radcr.2024.03.038. eCollection 2024 Jul.	Ton MD	2024
37228092	J Appl Lab Med. 2023 Jul 5;8(4):826-830. doi: 10.1093/jalm/jfad016.	Hernandez-Martinez V	2023
37109081	J Pers Med. 2023 Apr 20;13(4):695. doi: 10.3390/jpm13040695.	Sanz-Solas A	2023
37716912	Clin Pharmacol Ther. 2023 Dec;114(6):1350-1357. doi: 10.1002/cpt.3051. Epub 2023 Nov 2.	Lemke LK	2023
39822142	Clin Transl Sci. 2025 Jan;18(1):e70131. doi: 10.1111/cts.70131.	Tunehag KR	2025
39725012	J Mol Diagn. 2025 Mar;27(3):209-215. doi: 10.1016/j.jmoldx.2024.12.001. Epub 2024 Dec 24.	Burke KA	2025
39041736	Pharmacogenomics. 2024;25(7):289-291. doi: 10.1080/14622416.2024.2366691. Epub 2024 Jul 23.	Leach M	2024
36072879	Front Cardiovasc Med. 2022 Aug 22;9:994184. doi: 10.3389/fcvm.2022.994184. eCollection 2022.	Chen YW	2022
39338398	Pharmaceuticals (Basel). 2024 Sep 19;17(9):1236. doi: 10.3390/ph17091236.	Rodríguez-Lopez A	2024
36082121	Front Cardiovasc Med. 2022 Aug 23;9:991646. doi: 10.3389/fcvm.2022.991646. eCollection 2022.	Nguyen AB	2022
37713840	Medicine (Baltimore). 2023 Sep 15;102(37):e34974. doi: 10.1097/MD.0000000000034974.	Gao ST	2023
37528442	Clin Pharmacol Ther. 2023 Nov;114(5):1033-1042. doi: 10.1002/cpt.3012. Epub 2023 Aug 11.	Zubiaur P	2023
38440340	J Geriatr Cardiol. 2024 Jan 28;21(1):90-103. doi: 10.26599/1671-5411.2024.01.004.	Hu CY	2024
37083854	Mikrochim Acta. 2023 Apr 21;190(5):189. doi: 10.1007/s00604-023-05781-4.	Ni Y	2023
37383676	Pharmgenomics Pers Med. 2023 Jun 23;16:645-664. doi: 10.2147/PGPM.S371994. eCollection 2023.	Eken E	2023
37104082	Pharmacy (Basel). 2023 Apr 17;11(2):76. doi: 10.3390/pharmacy11020076.	Mir JF	2023
39691389	Front Pharmacol. 2024 Dec 3;15:1476765. doi: 10.3389/fphar.2024.1476765. eCollection 2024.	Youn MS	2024
38106031	medRxiv [Preprint]. 2023 Dec 7:2023.12.05.23299140. doi: 10.1101/2023.12.05.23299140.	Yang G	2023
39796117	Int J Mol Sci. 2024 Dec 30;26(1):260. doi: 10.3390/ijms26010260.	González-Iglesias E	2024
37808344	JACC Adv. 2023 Sep;2(7):None. doi: 10.1016/j.jacadv.2023.100573.	Magavern EF	2023

39681357	Ann Lab Med. 2025 Mar 1;45(2):121-132. doi: 10.3343/alm.2024.0572. Epub 2024 Dec 17.	Rim JH	2025
38737752	Clin Transl Discov. 2024 Jul;4(3):e296. doi: 10.1002/ctd2.296. Epub 2024 May 1.	Omidiran O	2024
40640196	NPJ Genom Med. 2025 Jul 10;10(1):54. doi: 10.1038/s41525-025-00511-6.	Shorbaji A	2025
40459439	Clin Pharmacol Ther. 2025 Oct;118(4):876-884. doi: 10.1002/cpt.3741. Epub 2025 Jun 3.	Chanfreau-Coffinier C	2025
39314259	Ther Adv Neurol Disord. 2024 Sep 19;17:17562864241273087. doi: 10.1177/17562864241273087. eCollection	Li Y	2024
39334336	BMC Pharmacol Toxicol. 2024 Sep 27;25(1):67. doi: 10.1186/s40360-024-00796-w.	Lan K	2024
39269241	Health Technol Assess. 2024 Sep;28(57):1-194. doi: 10.3310/PWCB4016.	Carroll J	2024
39150361	Clin Transl Sci. 2024 Aug;17(8):e70004. doi: 10.1111/cts.70004.	Cavallari LH	2024
38975048	Appl Clin Genet. 2024 Jul 2;17:95-105. doi: 10.2147/TACG.S463965. eCollection 2024.	Nakhonsri V	2024
35449399	Pharmacogenomics J. 2022 Dec;22(5-6):303-307. doi: 10.1038/s41397-022-00278-4. Epub 2022 Apr 21.	Baudhuin LM	2022
37551775	Clin Transl Sci. 2023 Oct;16(10):2010-2020. doi: 10.1111/cts.13608. Epub 2023 Aug 16.	Ramste M	2023
38699337	Res Sq [Preprint]. 2024 Apr 19:rs.3.rs-4152279. doi: 10.21203/rs.3.rs-4152279/v1.	Ike JI	2024
38700818	Dokl Biochem Biophys. 2024 Jun;516(1):83-92. doi: 10.1134/S1607672924600088. Epub 2024 May 3.	Fakhri YA	2024
38586400	Heliyon. 2024 Mar 27;10(7):e28566. doi: 10.1016/j.heliyon.2024.e28566. eCollection 2024 Apr 15.	Alonso Llorente A	2024
39640415	J Pharm Policy Pract. 2024 Dec 4;17(1):2432460. doi: 10.1080/20523211.2024.2432460. eCollection 2024.	Mason T	2024
39229818	Pharmacogenomics. 2024;25(8-9):407-423. doi: 10.1080/14622416.2024.2392479. Epub 2024 Sep 4.	Tomlinson E	2024
40070858	Balkan J Med Genet. 2025 Mar 6;27(2):77-85. doi: 10.2478/bjmg-2024-0015. eCollection 2024 Dec.	Elshani N	2025
38480590	J Thromb Thrombolysis. 2024 Apr;57(4):566-575. doi: 10.1007/s11239-024-02953-8. Epub 2024 Mar 13.	Gurbel PA	2024
40055373	NPJ Genom Med. 2025 Mar 7;10(1):20. doi: 10.1038/s41525-025-00479-3.	Yang G	2025
40710391	J Pers Med. 2025 Jun 27;15(7):274. doi: 10.3390/jpm15070274.	Nieh HV	2025
40926880	Res Pract Thromb Haemost. 2025 Aug 7;9(6):102997. doi: 10.1016/j.rpth.2025.102997. eCollection 2025 Aug.	Dang D	2025
38025166	Innov Pharm. 2023 Oct 10;14(2):10.24926/iip.v14i2.5476. doi: 10.24926/iip.v14i2.5476. eCollection 2023.	Bartlett B	2023
37601806	Int J Gen Med. 2023 Aug 14;16:3473-3481. doi: 10.2147/IJGM.S420108. eCollection 2023.	Shi Y	2023
38828314	Heliyon. 2024 May 18;10(11):e31383. doi: 10.1016/j.heliyon.2024.e31383. eCollection 2024 Jun 15.	Xiaoyong T	2024
37279000	JAMA Netw Open. 2023 Jun 1;6(6):e2317037. doi: 10.1001/jamanetworkopen.2023.17037.	Xie X	2023
37423841	Vet Clin North Am Small Anim Pract. 2023 Nov;53(6):1255-1276. doi: 10.1016/j.cvsm.2023.05.016. Epub 2023 J	Rivas VN	2023
39834800	Front Pharmacol. 2025 Jan 6;15:1484130. doi: 10.3389/fphar.2024.1484130. eCollection 2024.	Ren Z	2025
40465239	JAMA Surg. 2025 Jul 1;160(7):804-813. doi: 10.1001/jamasurg.2025.1503.	Tuteja S	2025
40280918	Pharmacogenomics J. 2025 Apr 25;25(3):12. doi: 10.1038/s41397-025-00371-4.	Patel TJ	2025
41068898	BMC Med Genomics. 2025 Oct 9;18(1):154. doi: 10.1186/s12920-025-02225-1.	Climacosa FMM	2025
41151929	J Clin Neurol. 2025 Nov;21(6):514-526. doi: 10.3988/jcn.2025.0317.	Park H	2025

36143168	J Pers Med. 2022 Aug 26;12(9):1383. doi: 10.3390/jpm12091383.	Pierre-François MJD	2022
38107326	Heliyon. 2023 Nov 11;9(12):e22214. doi: 10.1016/j.heliyon.2023.e22214. eCollection 2023 Dec.	Gao H	2023
38126046	J Cardiol Cases. 2023 Sep 1;28(6):265-268. doi: 10.1016/j.jccase.2023.08.011. eCollection 2023 Dec.	Hiraya D	2023
36803512	Expert Opin Drug Saf. 2023 Jan-Jun;22(6):477-484. doi: 10.1080/14740338.2023.2181333. Epub 2023 Feb 22.	Rodriguez-Monguio R	2023
35972991	Basic Clin Pharmacol Toxicol. 2022 Nov;131(5):311-324. doi: 10.1111/bcpt.13780. Epub 2022 Aug 24.	Iversen DB	2022
37693307	Front Genet. 2023 Aug 24;14:1242711. doi: 10.3389/fgene.2023.1242711. eCollection 2023.	Li G	2023
37449992	Eur J Clin Pharmacol. 2023 Sep;79(9):1249-1259. doi: 10.1007/s00228-023-03519-y. Epub 2023 Jul 14.	Wu Y	2023
37686303	Int J Mol Sci. 2023 Aug 31;24(17):13498. doi: 10.3390/ijms241713498.	Matišić V	2023
37361233	Front Pharmacol. 2023 Jun 8;14:1201906. doi: 10.3389/fphar.2023.1201906. eCollection 2023.	de Jong LM	2023
37303270	Clin Pharmacol Ther. 2023 Aug;114(2):275-287. doi: 10.1002/cpt.2957. Epub 2023 Jun 12.	Oni-Orisan A	2023
39519058	Int J Mol Sci. 2024 Oct 26;25(21):11505. doi: 10.3390/ijms252111505.	Peña-Martín MC	2024
39230622	Cardiovasc Drugs Ther. 2024 Dec;38(6):1079-1081. doi: 10.1007/s10557-024-07627-4. Epub 2024 Sep 4.	Shao D	2024
38874073	J Am Heart Assoc. 2024 Jun 18;13(12):e033791. doi: 10.1161/JAHA.123.033791. Epub 2024 Jun 14.	Tunehag KR	2024
40092094	Am J Transl Res. 2025 Feb 25;17(2):1470-1479. doi: 10.62347/SUNC9944. eCollection 2025.	Peng D	2025
40162622	Pharmacogenomics. 2025 Jan-Feb;26(1-2):31-37. doi: 10.1080/14622416.2025.2478810. Epub 2025 Mar 31.	Yuan HW	2025
40295977	BMC Cardiovasc Disord. 2025 Apr 28;25(1):330. doi: 10.1186/s12872-025-04768-8.	Haj Saleh N	2025
39953666	Clin Transl Sci. 2025 Feb;18(2):e70080. doi: 10.1111/cts.70080.	Biswas M	2025
38884958	Pharmacogenomics. 2024;25(7):293-298. doi: 10.1080/14622416.2024.2355862. Epub 2024 Jun 6.	Park JJ	2024
38671468	BMC Med Genomics. 2024 Apr 26;17(1):109. doi: 10.1186/s12920-024-01886-8.	Peng P	2024
37873439	medRxiv [Preprint]. 2023 Oct 2:2023.09.29.23296372. doi: 10.1101/2023.09.29.23296372.	Yang G	2023
41241757	Clin Transl Sci. 2025 Nov;18(11):e70406. doi: 10.1111/cts.70406.	Matthias J	2025
36220808	Transl Psychiatry. 2022 Oct 11;12(1):442. doi: 10.1038/s41398-022-02203-6.	Pinzón-Espinosa J	2022
38399346	Pharmaceutics. 2024 Feb 19;16(2):292. doi: 10.3390/pharmaceutics16020292.	Tatarūnas V	2024
36065758	Clin Transl Sci. 2022 Nov;15(11):2613-2624. doi: 10.1111/cts.13386. Epub 2022 Sep 6.	Campodónico DM	2022
39408985	Int J Mol Sci. 2024 Oct 3;25(19):10657. doi: 10.3390/ijms251910657.	Monero-Paredes M	2024
38058595	Ment Health Clin. 2023 Dec 1;13(6):276-288. doi: 10.9740/mhc.2023.12.276. eCollection 2023 Dec.	Leung JG	2023
37631355	Pharmaceutics. 2023 Aug 15;15(8):2141. doi: 10.3390/pharmaceutics15082141.	Rakicevic L	2023
38501281	Clin Transl Sci. 2024 Mar;17(3):e13772. doi: 10.1111/cts.13772.	Huh KY	2024
37310478	Eur J Clin Pharmacol. 2023 Aug;79(8):1107-1116. doi: 10.1007/s00228-023-03522-3. Epub 2023 Jun 13.	Lee YJ	2023
40055519	Nat Med. 2025 Mar;31(3):751-761. doi: 10.1038/s41591-025-03558-1. Epub 2025 Mar 7.	Palma-Martínez MJ	2025
35914768	Annu Rev Pharmacol Toxicol. 2023 Jan 20;63:211-229. doi: 10.1146/annurev-pharmtox-051921-092701. Epub 2	Castrichini M	2023

40667755	ACS Sens. 2025 Sep 26;10(9):6819-6827. doi: 10.1021/acssensors.5c01577. Epub 2025 Jul 16.	Serapinas S	2025
38023338	Eur Cardiol. 2023 Oct 31;18:e60. doi: 10.15420/ecr.2023.27. eCollection 2023.	Tan CK	2023
38028290	Radiol Case Rep. 2023 Nov 8;19(1):330-340. doi: 10.1016/j.radcr.2023.09.107. eCollection 2024 Jan.	Takahashi K	2023
36306392	Clin Pharmacol Ther. 2023 Mar;113(3):615-623. doi: 10.1002/cpt.2776. Epub 2022 Nov 19.	Cavallari LH	2023
38421234	Clin Transl Sci. 2024 Mar;17(3):e13737. doi: 10.1111/cts.13737.	Russell C	2024
37761953	Genes (Basel). 2023 Sep 17;14(9):1813. doi: 10.3390/genes14091813.	Monero-Paredes M	2023
38761068	J Am Heart Assoc. 2024 May 21;13(10):e032248. doi: 10.1161/JAHA.123.032248. Epub 2024 May 18.	Kim JH	2024
40005966	Pharmaceuticals (Basel). 2025 Jan 23;18(2):151. doi: 10.3390/ph18020151.	Calleja S	2025
37531788	Patient Educ Couns. 2023 Oct;115:107904. doi: 10.1016/j.pec.2023.107904. Epub 2023 Jul 17.	Doyle TA	2023
40271064	Front Pharmacol. 2025 Apr 9;16:1554370. doi: 10.3389/fphar.2025.1554370. eCollection 2025.	Wang H	2025
40685887	Basic Clin Pharmacol Toxicol. 2025 Aug;137(2):e70087. doi: 10.1111/bcpt.70087.	Axelsson MAB	2025
40722793	Biomedicines. 2025 Jul 14;13(7):1723. doi: 10.3390/biomedicines13071723.	Mitsis A	2025
40988928	Front Genet. 2025 Sep 8;16:1618105. doi: 10.3389/fgene.2025.1618105. eCollection 2025.	Ni P	2025
41011206	Pharmaceuticals (Basel). 2025 Sep 5;18(9):1335. doi: 10.3390/ph18091335.	Ortega-Ayala A	2025
36072880	Front Cardiovasc Med. 2022 Aug 22;9:925518. doi: 10.3389/fcvm.2022.925518. eCollection 2022.	Zhang M	2022
38000851	J Thromb Haemost. 2023 Dec;21(12):3317-3328. doi: 10.1016/j.jtha.2023.09.013.	Stanger L	2023
40014467	Pharmacogenet Genomics. 2025 Apr 1;35(3):116-118. doi: 10.1097/FPC.0000000000000557. Epub 2024 Dec 1	Brailsford J	2025
36353710	Pharmgenomics Pers Med. 2022 Nov 2;15:879-911. doi: 10.2147/PGPM.S338601. eCollection 2022.	Asiimwe IG	2022
40426982	Biomedicines. 2025 May 9;13(5):1156. doi: 10.3390/biomedicines13051156.	Ortega-Ayala A	2025
40568692	Aust Prescr. 2025 Jun;48(3):82-86. doi: 10.18773/austprescr.2025.021.	Stocker SL	2025
40895399	Pharmgenomics Pers Med. 2025 Aug 25;18:209-217. doi: 10.2147/PGPM.S529276. eCollection 2025.	Zhou B	2025
