# Supplementary material for: Limited Visibility and Perception of the Clinical Relevance of Clopidogrel Pharmacogenetics in Cardiology Literature
Source: Clin Transl Sci. 2026 May 11;19(5):e70584. doi: 10.1111/cts.70584 (PMC13160923; doi:10.1111/cts.70584)
Supplement: Supplementary file 3 — Data S2: cts70584‐sup‐0003‐DataS2.pdf. [file CTS-19-e70584-s001.pdf]

| Citations - 2013 CPIC Guidelines | | | |
|---|---|---|---|
| PMID | Citation | First Author | Publication Year |
| 36090856 | Front Neurol. 2022 Aug 24;13:887243. doi: 10.3389/fneur.2022.887243. eCollection 2022. | Enomoto Y | 2022 |
| 36368780 | Clin Lab Med. 2022 Dec;42(4):533-546. doi: 10.1016/j.cll.2022.09.009. | Cavallari LH | 2022 |
| 27757066 | Dialogues Clin Neurosci. 2016 Sep;18(3):323-337. doi: 10.31887/DCNS.2016.18.3/jbishop. | Eum S | 2016 |
| 38207129 | Genome Biol Evol. 2024 Jan 5;16(1):evad236. doi: 10.1093/gbe/evad236. | Richard-St-Hilaire A | 2024 |
| 27573042 | Neth Heart J. 2016 Oct;24(10):589-99. doi: 10.1007/s12471-016-0873-z. | Deiman BA | 2016 |
| 32796505 | J Pers Med. 2020 Aug 11;10(3):84. doi: 10.3390/jpm10030084. | Thomas RE | 2020 |
| 36068466 | Methods Mol Biol. 2022;2547:201-240. doi: 10.1007/978-1-0716-2573-6_9. | Babayeva M | 2022 |
| 27214750 | Clin Transl Sci. 2016 Oct;9(5):233-245. doi: 10.1111/cts.12404. Epub 2016 Jun 14. | Arwood MJ | 2016 |
| 31504375 | Eur Heart J Cardiovasc Pharmacother. 2020 Jul 1;6(4):203-210. doi: 10.1093/ehjcvp/pvz045. | Lewis JP | 2020 |
| 36839726 | Pharmaceutics. 2023 Jan 25;15(2):404. doi: 10.3390/pharmaceutics15020404. | Soria-Chacartegui P | 2023 |
| 37894954 | Int J Mol Sci. 2023 Oct 17;24(20):15265. doi: 10.3390/ijms242015265. | Soria-Chacartegui P | 2023 |
| 32506666 | Clin Transl Sci. 2020 Nov;13(6):1298-1306. doi: 10.1111/cts.12830. Epub 2020 Jul 21. | Ionova Y | 2020 |
| 24510446 | Pharmacotherapy. 2014 Jul;34(7):703-18. doi: 10.1002/phar.1400. Epub 2014 Feb 7. | Owusu Obeng A | 2014 |
| 25381554 | Med Sci Monit. 2014 Nov 9;20:2213-8. doi: 10.12659/MSM.892475. | Li Y | 2014 |
| 25210416 | P T. 2014 Sep;39(9):630-7. | Westervelt P | 2014 |
| 26635887 | Per Med. 2015;12(4):339-347. doi: 10.2217/pme.15.10. | Unertl KM | 2015 |
| 33568995 | Front Pharmacol. 2021 Jan 25;11:595219. doi: 10.3389/fphar.2020.595219. eCollection 2020. | Abdullah-Koolmees H | 2021 |
| 38550953 | Camb Prism Precis Med. 2023 Jun 29;1:e28. doi: 10.1017/pcm.2023.17. eCollection 2023. | Padmanabhan S | 2023 |
| 29749904 | Per Med. 2016 Mar;13(2):119-127. doi: 10.2217/pme.15.57. Epub 2016 Mar 1. | Luzum JA | 2016 |
| 35094598 | Ann Pharmacother. 2022 Oct;56(10):1159-1173. doi: 10.1177/10600280211073009. Epub 2022 Jan | Kamarova M | 2022 |
| 27643672 | OMICS. 2016 Oct;20(10):593-603. doi: 10.1089/omi.2016.0122. Epub 2016 Sep 19. | Alessandrini M | 2016 |
| 25562653 | J Pers Med. 2013 Aug 21;3(3):203-37. doi: 10.3390/jpm3030203. | Gladding PA | 2013 |
| 32723143 | Pharmacogenomics. 2020 Aug;21(12):889-897. doi: 10.2217/pgs-2020-0046. Epub 2020 Jul 29. | Ellithi M | 2020 |
| 33085221 | Clin Transl Sci. 2021 Jan;14(1):153-162. doi: 10.1111/cts.12919. Epub 2020 Nov 22. | Stevenson JM | 2021 |
| 32662547 | J Clin Pharm Ther. 2020 Dec;45(6):1457-1465. doi: 10.1111/jcpt.13223. Epub 2020 Jul 14. | Nguyen TT | 2020 |
| 33023029 | J Pers Med. 2020 Oct 3;10(4):154. doi: 10.3390/jpm10040154. | Pasternak AL | 2020 |
| 38371288 | JACC Asia. 2024 Jan 23;4(2):135-137. doi: 10.1016/j.jacasi.2023.11.008. eCollection 2024 Feb. | Patel AP | 2024 |
| 30049953 | Pharmaceuticals (Basel). 2018 Jul 26;11(3):74. doi: 10.3390/ph11030074. | Zakaria ZH | 2018 |
| 35496350 | Pharmgenomics Pers Med. 2022 Apr 23;15:409-427. doi: 10.2147/PGPM.S348851. eCollection 2022 | Hayashi M | 2022 |

| | | | |
|---|---|---|---|
| 34683865 | Pharmaceutics. 2021 Sep 28;13(10):1573. doi: 10.3390/pharmaceutics13101573. | Zubiaur P | 2021 |
| 35614463 | J Pharm Policy Pract. 2022 May 25;15(1):39. doi: 10.1186/s40545-022-00435-x. | Abubakar U | 2022 |
| 35901007 | PLoS One. 2022 Jul 28;17(7):e0272140. doi: 10.1371/journal.pone.0272140. eCollection 2022. | Kitahara H | 2022 |
| 29858578 | Genet Med. 2019 Feb;21(2):382-390. doi: 10.1038/s41436-018-0057-x. Epub 2018 Jun 1. | Vassy JL | 2019 |
| 25421045 | Circulation. 2014 Nov 25;130(22):1971-80. doi: 10.1161/CIRCULATIONAHA.114.006511. | George AL Jr | 2014 |
| 29701129 | Pharmacogenomics. 2018 May;19(7):621-628. doi: 10.2217/pgs-2018-0013. Epub 2018 Apr 27. | Bergmeijer TO | 2018 |
| 28686612 | PLoS One. 2017 Jul 7;12(7):e0175508. doi: 10.1371/journal.pone.0175508. eCollection 2017. | Wei WQ | 2017 |
| 27981572 | Clin Pharmacol Ther. 2017 Jul;102(1):45-51. doi: 10.1002/cpt.583. Epub 2017 Apr 18. | Moriyama B | 2017 |
| 28546997 | Mol Genet Genomic Med. 2017 Mar 19;5(3):269-279. doi: 10.1002/mgg3.283. eCollection 2017 May. | Cousin MA | 2017 |
| 28612093 | Paediatr Drugs. 2017 Oct;19(5):379-389. doi: 10.1007/s40272-017-0244-2. | Balevic SJ | 2017 |
| 37310478 | Eur J Clin Pharmacol. 2023 Aug;79(8):1107-1116. doi: 10.1007/s00228-023-03522-3. Epub 2023 Jun | Lee YJ | 2023 |
| 33421282 | Clin Transl Sci. 2021 May;14(3):784-790. doi: 10.1111/cts.12973. Epub 2021 Feb 12. | Oni-Orisan A | 2021 |
| 33370281 | PLoS One. 2020 Dec 28;15(12):e0236260. doi: 10.1371/journal.pone.0236260. eCollection 2020. | Rath CL | 2020 |
| 30520344 | Pharmacogenomics. 2019 Jan;20(2):75-83. doi: 10.2217/pgs-2018-0148. Epub 2018 Dec 6. | Melin K | 2019 |
| 40421189 | Front Cardiovasc Med. 2025 May 12;12:1572389. doi: 10.3389/fcvm.2025.1572389. eCollection 202 | Wang Y | 2025 |
| 40802150 | Am J Cardiovasc Drugs. 2025 Nov;25(6):735-737. doi: 10.1007/s40256-025-00758-5. Epub 2025 Aug | Akbar MA | 2025 |
| 32759249 | BMJ Open. 2020 Aug 5;10(8):e038031. doi: 10.1136/bmjopen-2020-038031. | Song TJ | 2020 |
| 34037732 | JAMA Netw Open. 2021 May 3;4(5):e2110446. doi: 10.1001/jamanetworkopen.2021.10446. | Cohn I | 2021 |
| 26024717 | Int J Clin Pharm. 2015 Oct;37(5):925-30. doi: 10.1007/s11096-015-0143-y. Epub 2015 May 30. | Chawla PK | 2015 |
| 27551817 | Genet Test Mol Biomarkers. 2016 Oct;20(10):609-615. doi: 10.1089/gtmb.2016.0001. Epub 2016 Aug | Jaja C | 2016 |
| 32725974 | Korean Circ J. 2020 Aug;50(8):645-657. doi: 10.4070/kcj.2020.0098. | Cho H | 2020 |
| 26070947 | Clin Pharmacokinet. 2015 Dec;54(12):1223-35. doi: 10.1007/s40262-015-0297-8. | Elewa H | 2015 |
| 26272307 | Curr Hypertens Rep. 2015 Sep;17(9):586. doi: 10.1007/s11906-015-0586-5. | Arwood MJ | 2015 |
| 30188374 | Pharmacogenet Genomics. 2018 Sep;28(9):207-213. doi: 10.1097/FPC.0000000000000349. | Fathy S | 2018 |
| 36927865 | Nat Commun. 2023 Mar 17;14(1):1474. doi: 10.1038/s41467-023-37209-1. | Powell NR | 2023 |
| 35140503 | Pharmgenomics Pers Med. 2022 Feb 2;15:81-89. doi: 10.2147/PGPM.S335860. eCollection 2022. | Liao YJ | 2022 |
| 36970128 | Res Pract Thromb Haemost. 2023 Feb 24;7(2):100093. doi: 10.1016/j.rpth.2023.100093. eCollection | Song PY | 2023 |
| 35600119 | Inf Serv Use. 2022 May 10;42(1):71-80. doi: 10.3233/ISU-210144. eCollection 2022. | Mo H | 2022 |
| 34782755 | Eur J Hum Genet. 2022 Oct;30(10):1114-1120. doi: 10.1038/s41431-021-01004-7. Epub 2021 Nov 16 | Brouwer JMJL | 2022 |
| 31086207 | Sci Rep. 2019 May 13;9(1):7323. doi: 10.1038/s41598-019-43736-z. | Afsar NA | 2019 |
| 32128178 | Clin Case Rep. 2019 Dec 23;8(2):305-312. doi: 10.1002/ccr3.2604. eCollection 2020 Feb. | Bain KT | 2019 |

| | | | |
|---|---|---|---|
| 24743898 | Curr Cardiol Rep. 2014;16(6):488. doi: 10.1007/s11886-014-0488-1. | Abraham G | 2014 |
| 26757134 | Clin Transl Sci. 2016 Feb;9(1):23-8. doi: 10.1111/cts.12383. Epub 2016 Jan 12. | Khalil BM | 2016 |
| 26980150 | Int J Clin Pharm. 2016 Apr;38(2):414-20. doi: 10.1007/s11096-016-0269-6. Epub 2016 Mar 15. | Wirth F | 2016 |
| 28685396 | AAPS J. 2017 Sep;19(5):1513-1522. doi: 10.1208/s12248-017-0102-8. Epub 2017 Jul 6. | Connarn JN | 2017 |
| 28277330 | Pharmacogenet Genomics. 2017 May;27(5):201-209. doi: 10.1097/FPC.0000000000000276. | Barbarino JM | 2017 |
| 26522758 | Ann Lab Med. 2016 Jan;36(1):42-8. doi: 10.3343/alm.2016.36.1.42. | Li S | 2016 |
| 28203101 | Pharmgenomics Pers Med. 2017 Jan 27;10:17-27. doi: 10.2147/PGPM.S123719. eCollection 2017. | Daneshi N | 2017 |
| 28294551 | Clin Transl Sci. 2017 May;10(3):143-146. doi: 10.1111/cts.12456. Epub 2017 Mar 14. | Cavallari LH | 2017 |
| 26447442 | Am Fam Physician. 2015 Oct 1;92(7):588-94. | Chang KL | 2015 |
| 28029011 | Ann Lab Med. 2017 Mar;37(2):180-193. doi: 10.3343/alm.2017.37.2.180. | Kim S | 2017 |
| 28982267 | Pharmacogenomics. 2017 Oct;18(15):1381-1386. doi: 10.2217/pgs-2017-0137. Epub 2017 Oct 6. | Scott SA | 2017 |
| 28815127 | AMIA Jt Summits Transl Sci Proc. 2017 Jul 26;2017:175-184. eCollection 2017. | Lee CJ | 2017 |
| 24398597 | Clin Pharmacol Ther. 2014 Apr;95(4):394-402. doi: 10.1038/clpt.2013.226. Epub 2014 Feb 19. | Dotson WD | 2014 |
| 37306816 | Handb Exp Pharmacol. 2023;280:3-32. doi: 10.1007/164_2023_658. | McDermott JH | 2023 |
| 39245547 | Interv Cardiol Clin. 2024 Oct;13(4):469-481. doi: 10.1016/j.iccl.2024.06.002. Epub 2024 Aug 1. | Cavallari LH | 2024 |
| 32916938 | Cells. 2020 Sep 9;9(9):2056. doi: 10.3390/cells9092056. | Nassar SF | 2020 |
| 32708920 | J Clin Med. 2020 Jul 17;9(7):2274. doi: 10.3390/jcm9072274. | Arwood MJ | 2020 |
| 27665573 | Clin Pharmacokinet. 2017 May;56(5):525-536. doi: 10.1007/s40262-016-0454-8. | Green B | 2017 |
| 32683419 | Pharmacogenomics J. 2021 Feb;21(1):47-59. doi: 10.1038/s41397-020-0175-0. Epub 2020 Jul 18. | Ingram CJE | 2021 |
| 35402528 | Front Cardiovasc Med. 2022 Mar 23;9:850028. doi: 10.3389/fcvm.2022.850028. eCollection 2022. | Al-Abcha A | 2022 |
| 30106264 | Neuropsychopharmacol Rep. 2018 Mar;38(1):2-8. doi: 10.1002/npr2.12003. Epub 2018 Feb 6. | Yoshida K | 2018 |
| 35156424 | J Am Heart Assoc. 2022 Feb 15;11(4):e024159. doi: 10.1161/JAHA.121.024159. Epub 2022 Feb 12. | Beitelshees AL | 2022 |
| 30506572 | Clin Pharmacol Ther. 2019 May;105(5):1256-1262. doi: 10.1002/cpt.1322. Epub 2019 Jan 21. | Huddart R | 2019 |
| 31197411 | Eur J Clin Pharmacol. 2019 Sep;75(9):1201-1210. doi: 10.1007/s00228-019-02696-z. Epub 2019 Jun | Janssen PWA | 2019 |
| 29385765 | J Pers Med. 2018 Jan 30;8(1):8. doi: 10.3390/jpm8010008. | Brown SA | 2018 |
| 32267573 | Br J Clin Pharmacol. 2020 Sep;86(9):1860-1874. doi: 10.1111/bcp.14296. Epub 2020 Jun 17. | Zhang Y | 2020 |
| 29347970 | BMC Res Notes. 2018 Jan 18;11(1):46. doi: 10.1186/s13104-018-3132-0. | Hassani Idrissi H | 2018 |
| 25243032 | Pharm Pract (Granada). 2014 Jul;12(3):438. doi: 10.4321/s1886-36552014000300007. Epub 2014 Se | Patel V | 2014 |
| 24803100 | Br J Clin Pharmacol. 2014 Nov;78(5):1058-66. doi: 10.1111/bcp.12416. | Siepmann T | 2014 |
| 26958179 | AMIA Annu Symp Proc. 2015 Nov 5;2015:466-74. eCollection 2015. | Cutting EM | 2015 |
| 34471538 | SAGE Open Med. 2021 Aug 26;9:20503121211042209. doi: 10.1177/20503121211042209. eCollect | Islam MR | 2021 |

| | | | |
|---|---|---|---|
| 26693963 | Clin Pharmacol Ther. 2016 Jul;100(1):67-74. doi: 10.1002/cpt.331. Epub 2016 Feb 17. | Peterson JF | 2016 |
| 27864202 | Am J Health Syst Pharm. 2016 Dec 1;73(23):1944-1954. doi: 10.2146/ajhp150946. | Cavallari LH | 2016 |
| 38790236 | Genes (Basel). 2024 May 10;15(5):607. doi: 10.3390/genes15050607. | Ganoci L | 2024 |
| 28385198 | Mayo Clin Proc. 2017 Apr;92(4):642-662. doi: 10.1016/j.mayocp.2017.01.015. | Giudicessi JR | 2017 |
| 28335443 | Int J Environ Res Public Health. 2017 Mar 14;14(3):301. doi: 10.3390/ijerph14030301. | Zhang YJ | 2017 |
| 28594278 | Pharmacogenomics. 2017 Jun;18(9):835-841. doi: 10.2217/pgs-2017-0042. Epub 2017 Jun 8. | Pierson RC | 2017 |
| 33315113 | JAMA Netw Open. 2020 Dec 1;3(12):e2029411. doi: 10.1001/jamanetworkopen.2020.29411. | Ramsey LB | 2020 |
| 29199461 | Exp Biol Med (Maywood). 2018 Feb;243(3):313-322. doi: 10.1177/1535370217744775. Epub 2017 De | Wilson JL | 2018 |
| 33145028 | NPJ Genom Med. 2020 Oct 30;5:48. doi: 10.1038/s41525-020-00156-7. eCollection 2020. | Shugg T | 2020 |
| 35657416 | Eur J Clin Pharmacol. 2022 Aug;78(8):1217-1225. doi: 10.1007/s00228-022-03346-7. Epub 2022 Jun | Huang S | 2022 |
| 40916919 | Pharmacotherapy. 2025 Oct;45(10):654-666. doi: 10.1002/phar.70058. Epub 2025 Sep 8. | Jeong E | 2025 |
| 33829662 | Mol Genet Genomic Med. 2021 Jun;9(6):e1680. doi: 10.1002/mgg3.1680. Epub 2021 Apr 7. | Cutrer FM | 2021 |
| 26196021 | Open Heart. 2015 Jun 30;2(1):e000248. doi: 10.1136/openhrt-2015-000248. eCollection 2015. | Cardoso RN | 2015 |
| 32702814 | Medicine (Baltimore). 2020 Jul 17;99(29):e20582. doi: 10.1097/MD.0000000000020582. | Wang T | 2020 |
| 26030725 | Genet Res (Camb). 2015 Jun 1;97:e13. doi: 10.1017/S0016672315000099. | Hess GP | 2015 |
| 36160382 | Front Pharmacol. 2022 Sep 8;13:952804. doi: 10.3389/fphar.2022.952804. eCollection 2022. | Ye Z | 2022 |
| 33817306 | Open Life Sci. 2021 Feb 18;16(1):142-149. doi: 10.1515/biol-2021-0017. eCollection 2021. | Mugosa S | 2021 |
| 31327749 | J Am Pharm Assoc (2003). 2019 Sep-Oct;59(5):646-650. doi: 10.1016/j.japh.2019.06.008. Epub 2019 | Gammal RS | 2019 |
| 34984913 | Per Med. 2022 Mar;19(2):93-101. doi: 10.2217/pme-2021-0064. Epub 2022 Jan 5. | Lee G | 2022 |
| 30930780 | Front Pharmacol. 2019 Mar 14;10:240. doi: 10.3389/fphar.2019.00240. eCollection 2019. | Hočevar K | 2019 |
| 34652573 | Hum Genet. 2022 Jun;141(6):1113-1136. doi: 10.1007/s00439-021-02385-x. Epub 2021 Oct 15. | Zhou Y | 2022 |
| 32158254 | Pharmgenomics Pers Med. 2020 Jan 23;13:29-37. doi: 10.2147/PGPM.S234910. eCollection 2020. | Sychev DA | 2020 |
| 34834577 | J Pers Med. 2021 Nov 18;11(11):1226. doi: 10.3390/jpm11111226. | O'Brien TJ | 2021 |
| 32014855 | Cold Spring Harb Mol Case Stud. 2020 Feb 3;6(1):a004531. doi: 10.1101/mcs.a004531. Print 2020 Fe | Wilk MA | 2020 |
| 32355288 | Cell Res. 2020 Sep;30(9):717-731. doi: 10.1038/s41422-020-0322-9. Epub 2020 Apr 30. | Cao Y | 2020 |
| 25123743 | Yearb Med Inform. 2014 Aug 15;9(1):199-205. doi: 10.15265/IY-2014-0015. | Denny JC | 2014 |
| 37342754 | Cureus. 2023 May 20;15(5):e39265. doi: 10.7759/cureus.39265. eCollection 2023 May. | Gairolla J | 2023 |
| 29123971 | Curr Transplant Rep. 2017 Jun;4(2):116-123. doi: 10.1007/s40472-017-0153-x. Epub 2017 May 24. | Oetting WS | 2017 |
| 33638977 | Eur Heart J Cardiovasc Pharmacother. 2022 Jan 5;8(1):85-99. doi: 10.1093/ehjcvp/pvab018. | Magavern EF | 2022 |
| 33048353 | Clin Pharmacol Ther. 2021 Jan;109(1):101-115. doi: 10.1002/cpt.2079. Epub 2020 Nov 15. | Liu M | 2021 |
| 36107166 | Genet Med. 2022 Nov;24(11):2338-2350. doi: 10.1016/j.gim.2022.08.009. Epub 2022 Sep 15. | Lau-Min KS | 2022 |

| | | | |
|---|---|---|---|
| 37963685 | Open Heart. 2023 Nov;10(2):e002436. doi: 10.1136/openhrt-2023-002436. | Lopez J | 2023 |
| 27686864 | Sci Rep. 2016 Sep 30;6:34570. doi: 10.1038/srep34570. | Li XG | 2016 |
| 32821149 | Pharmgenomics Pers Med. 2020 Jul 27;13:239-252. doi: 10.2147/PGPM.S231475. eCollection 2020. | Gower MN | 2020 |
| 32897581 | Clin Pharmacol Ther. 2021 Mar;109(3):705-715. doi: 10.1002/cpt.2039. Epub 2020 Oct 2. | Lee CR | 2021 |
| 35449399 | Pharmacogenomics J. 2022 Dec;22(5-6):303-307. doi: 10.1038/s41397-022-00278-4. Epub 2022 Ap | Baudhuin LM | 2022 |
| 30296897 | Annu Rev Pharmacol Toxicol. 2019 Jan 6;59:577-603. doi: 10.1146/annurev-pharmtox-010818-02115 | De T | 2019 |
| 35629210 | J Pers Med. 2022 May 13;12(5):788. doi: 10.3390/jpm12050788. | Ovejero-Benito MC | 2022 |
| 37511655 | J Pers Med. 2023 Jun 25;13(7):1042. doi: 10.3390/jpm13071042. | Ghasim H | 2023 |
| 30939847 | Genes (Basel). 2019 Apr 1;10(4):261. doi: 10.3390/genes10040261. | Dávila-Fajardo CL | 2019 |
| 37763014 | J Clin Med. 2023 Sep 20;12(18):6074. doi: 10.3390/jcm12186074. | Nardin M | 2023 |
| 24668607 | Curr Cardiol Rep. 2014 May;16(5):485. doi: 10.1007/s11886-014-0485-4. | Jeong YH | 2014 |
| 32109992 | Drug Des Devel Ther. 2020 Feb 19;14:669-676. doi: 10.2147/DDDT.S242167. eCollection 2020. | Zhang M | 2020 |
| 31963461 | Int J Mol Sci. 2020 Jan 17;21(2):604. doi: 10.3390/ijms21020604. | Fatunde OA | 2020 |
| 34154743 | Indian Heart J. 2021 May-Jun;73(3):281-288. doi: 10.1016/j.ihj.2021.03.004. Epub 2021 Mar 17. | Stys TP | 2021 |
| 38806977 | Curr Cardiol Rep. 2024 Jul;26(7):675-680. doi: 10.1007/s11886-024-02071-0. Epub 2024 May 28. | Rajachandran M | 2024 |
| 37274118 | Front Pharmacol. 2023 May 18;14:1189976. doi: 10.3389/fphar.2023.1189976. eCollection 2023. | Kabbani D | 2023 |
| 34596891 | Transl Stroke Res. 2022 Jun;13(3):391-398. doi: 10.1007/s12975-021-00949-7. Epub 2021 Oct 1. | Lv H | 2022 |
| 28124392 | Clin Pharmacol Ther. 2017 Sep;102(3):493-501. doi: 10.1002/cpt.631. Epub 2017 Jun 1. | Harada S | 2017 |
| 31664775 | Clin Transl Sci. 2020 Mar;13(2):260-264. doi: 10.1111/cts.12714. Epub 2019 Nov 29. | Davis BH | 2020 |
| 39365028 | Clin Pharmacol Ther. 2025 Jan;117(1):278-288. doi: 10.1002/cpt.3458. Epub 2024 Oct 4. | Litonius K | 2025 |
| 39258919 | Pharmacogenomics. 2024;25(8-9):391-399. doi: 10.1080/14622416.2024.2394014. Epub 2024 Sep | Massmann A | 2024 |
| 39360670 | Pharmacogenomics. 2024;25(12-13):503-513. doi: 10.1080/14622416.2024.2406213. Epub 2024 O | Fox LP | 2024 |
| 26021633 | Future Cardiol. 2015 May;11(3):281-6. doi: 10.2217/fca.15.20. | Cavallari LH | 2015 |
| 36340250 | JACC Asia. 2022 Feb 15;2(1):1-18. doi: 10.1016/j.jacasi.2021.12.005. eCollection 2022 Feb. | Kwon O | 2022 |
| 25974703 | Clin Pharmacol Ther. 2015 Aug;98(2):127-34. doi: 10.1002/cpt.147. Epub 2015 Jun 29. | Hicks JK | 2015 |
| 31394823 | J Pers Med. 2019 Aug 7;9(3):40. doi: 10.3390/jpm9030040. | Hippman C | 2019 |
| 34834403 | J Pers Med. 2021 Oct 20;11(11):1051. doi: 10.3390/jpm11111051. | Liu M | 2021 |
| 29615455 | Circ Genom Precis Med. 2018 Apr;11(4):e002118. doi: 10.1161/CIRCGEN.118.002118. | Lewis JP | 2018 |
| 25292429 | Annu Rev Pharmacol Toxicol. 2015;55:89-106. doi: 10.1146/annurev-pharmtox-010814-124835. Epu | Dunnenberger HM | 2015 |
| 34222372 | Front Cardiovasc Med. 2021 Jun 16;8:676954. doi: 10.3389/fcvm.2021.676954. eCollection 2021. | Shi X | 2021 |
| 38702595 | BMC Pediatr. 2024 May 3;24(1):299. doi: 10.1186/s12887-024-04625-1. | Zhao T | 2024 |

| | | | |
|---|---|---|---|
| 28597175 | Int J Clin Pharm. 2017 Aug;39(4):791-797. doi: 10.1007/s11096-017-0451-5. Epub 2017 Jun 9. | Li J | 2017 |
| 34616423 | Front Genet. 2021 Sep 20;12:687116. doi: 10.3389/fgene.2021.687116. eCollection 2021. | Nagaraj SH | 2021 |
| 33246208 | J Stroke Cerebrovasc Dis. 2021 Feb;30(2):105464. doi: 10.1016/j.jstrokecerebrovasdis.2020.105464 | Patel PD | 2021 |
| 33551797 | Front Pharmacol. 2021 Jan 20;11:582929. doi: 10.3389/fphar.2020.582929. eCollection 2020. | Zhang Y | 2021 |
| 27798644 | PLoS One. 2016 Oct 31;11(10):e0164169. doi: 10.1371/journal.pone.0164169. eCollection 2016. | Ang GY | 2016 |
| 33707344 | Aging (Albany NY). 2021 Mar 12;13(5):6506-6524. doi: 10.18632/aging.202799. Epub 2021 Mar 12. | Cha JJ | 2021 |
| 35307978 | Pharmacol Res Perspect. 2022 Apr;10(2):e00946. doi: 10.1002/prp2.946. | Duong JK | 2022 |
| 35155618 | Front Cardiovasc Med. 2022 Jan 27;8:818215. doi: 10.3389/fcvm.2021.818215. eCollection 2021. | Sun M | 2022 |
| 35570383 | Med Sci Monit. 2022 May 16;28:e935664. doi: 10.12659/MSM.935664. | Tao L | 2022 |
| 29384027 | J Manag Care Spec Pharm. 2018 Feb;24(2):142-152. doi: 10.18553/jmcp.2018.24.2.142. | Okere AN | 2018 |
| 30901549 | Cell. 2019 Mar 21;177(1):58-69. doi: 10.1016/j.cell.2019.02.039. | Abul-Husn NS | 2019 |
| 35089958 | PLoS One. 2022 Jan 28;17(1):e0263137. doi: 10.1371/journal.pone.0263137. eCollection 2022. | Goljan E | 2022 |
| 30910893 | Antimicrob Agents Chemother. 2019 May 24;63(6):e02399-18. doi: 10.1128/AAC.02399-18. Print 20 | Bernal-Martínez L | 2019 |
| 26444257 | Pharmacogenet Genomics. 2016 Jan;26(1):28-39. doi: 10.1097/FPC.0000000000000182. | Chan SL | 2016 |
| 37081514 | Eur J Med Res. 2023 Apr 21;28(1):154. doi: 10.1186/s40001-023-01115-5. | Deng T | 2023 |
| 29263831 | NPJ Genom Med. 2017 May 26;2:19. doi: 10.1038/s41525-017-0021-8. eCollection 2017. | Cohn I | 2017 |
| 33578832 | Metabolites. 2021 Feb 10;11(2):96. doi: 10.3390/metabo11020096. | Westergaard N | 2021 |
| 33352764 | Pharmaceutics. 2020 Dec 19;12(12):1240. doi: 10.3390/pharmaceutics12121240. | Malsagova KA | 2020 |
| 27528039 | Drug Metab Dispos. 2016 Nov;44(11):1832-1838. doi: 10.1124/dmd.116.071530. Epub 2016 Aug 15. | Fokina VM | 2016 |
| 31871135 | Drug Metab Dispos. 2020 Mar;48(3):230-244. doi: 10.1124/dmd.119.089680. Epub 2019 Dec 23. | Her L | 2020 |
| 39933830 | Open Heart. 2025 Feb 11;12(1):e003088. doi: 10.1136/openhrt-2024-003088. | Pareek A | 2025 |
| 35197312 | Drug Metab Dispos. 2023 Jan;51(1):29-37. doi: 10.1124/dmd.121.000624. Epub 2022 Feb 23. | Perez-Paramo YX | 2023 |
| 33993817 | Expert Rev Clin Pharmacol. 2021 Aug;14(8):963-978. doi: 10.1080/17512433.2021.1927709. Epub 2 | Galli M | 2021 |
| 26071289 | Indian Heart J. 2015 Mar-Apr;67(2):114-21. doi: 10.1016/j.ihj.2015.03.017. Epub 2015 Apr 27. | Rath PC | 2015 |
| 27112273 | BMC Fam Pract. 2016 Apr 26;17:49. doi: 10.1186/s12875-016-0447-6. | Stingl JC | 2016 |
| 31807051 | Pharmgenomics Pers Med. 2019 Nov 5;12:329-339. doi: 10.2147/PGPM.S217725. eCollection 2019. | Dorofeeva MN | 2019 |
| 31807435 | Integr Pharm Res Pract. 2019 Nov 8;8:97-104. doi: 10.2147/IPRP.S180154. eCollection 2019. | Elewa H | 2019 |
| 35197553 | Pharmacogenomics J. 2022 May;22(3):166-172. doi: 10.1038/s41397-022-00270-y. Epub 2022 Feb 2 | Häkkinen K | 2022 |
| 30137643 | Clin Pharmacol Ther. 2019 Aug;106(2):360-373. doi: 10.1002/cpt.1223. Epub 2018 Oct 18. | Vassy JL | 2019 |
| 31385522 | Future Cardiol. 2019 Jul;15(4):251-254. doi: 10.2217/fca-2019-0017. Epub 2019 Aug 6. | Cavallari LH | 2019 |
| 37895189 | Genes (Basel). 2023 Sep 22;14(10):1841. doi: 10.3390/genes14101841. | van der Drift D | 2023 |

| | | | |
|---|---|---|---|
| 32236952 | Clin Pharmacol Ther. 2020 Aug;108(2):338-349. doi: 10.1002/cpt.1834. Epub 2020 Apr 23. | Maliepaard M | 2020 |
| 29642909 | J Transl Med. 2018 Apr 11;16(1):92. doi: 10.1186/s12967-018-1469-8. | Cavallari LH | 2018 |
| 32343201 | Pharmacogenomics. 2020 May;21(7):431-441. doi: 10.2217/pgs-2019-0185. Epub 2020 Apr 28. | Black RM | 2020 |
| 29488032 | Curr Treat Options Cardiovasc Med. 2018 Feb 27;20(2):17. doi: 10.1007/s11936-018-0603-5. | Mangels DR | 2018 |
| 24867283 | Drug Metabol Drug Interact. 2014;29(4):221-33. doi: 10.1515/dmdi-2013-0069. | Yang Y | 2014 |
| 34384383 | BMC Cardiovasc Disord. 2021 Aug 12;21(1):391. doi: 10.1186/s12872-021-02201-4. | Yu L | 2021 |
| 34415683 | Clin Transl Sci. 2021 Nov;14(6):2254-2266. doi: 10.1111/cts.13124. Epub 2021 Aug 20. | Duconge J | 2021 |
| 38529185 | Front Pharmacol. 2024 Mar 11;15:1349203. doi: 10.3389/fphar.2024.1349203. eCollection 2024. | Gan P | 2024 |
| 26979147 | Adv Chronic Kidney Dis. 2016 Mar;23(2):82-90. doi: 10.1053/j.ackd.2015.12.001. | Cavallari LH | 2016 |
| 28689434 | Expert Rev Clin Pharmacol. 2018 Feb;11(2):151-164. doi: 10.1080/17512433.2017.1353909. Epub 2 | Moon JY | 2018 |
| 29057167 | Curr Genet Med Rep. 2016 Sep;4(3):119-129. doi: 10.1007/s40142-016-0096-z. Epub 2016 Jun 16. | Tuteja S | 2016 |
| 28699807 | Expert Rev Cardiovasc Ther. 2017 Aug;15(8):581-589. doi: 10.1080/14779072.2017.1355236. Epub 2 | Cavallari LH | 2017 |
| 26725101 | Am J Cardiol. 2016 Feb 15;117(4):656-663. doi: 10.1016/j.amjcard.2015.11.029. Epub 2015 Dec 7. | Jain N | 2016 |
| 28046094 | PLoS One. 2017 Jan 3;12(1):e0169233. doi: 10.1371/journal.pone.0169233. eCollection 2017. | Goh LL | 2017 |
| 26021325 | Drug Metab Dispos. 2015 Aug;43(8):1226-35. doi: 10.1124/dmd.115.064428. Epub 2015 May 28. | Chaudhry AS | 2015 |
| 38480590 | J Thromb Thrombolysis. 2024 Apr;57(4):566-575. doi: 10.1007/s11239-024-02953-8. Epub 2024 Mar | Gurbel PA | 2024 |
| 41158875 | Front Mol Biosci. 2025 Oct 13;12:1669085. doi: 10.3389/fmolb.2025.1669085. eCollection 2025. | Wagner JT | 2025 |
| 23872826 | Clin Pharmacol Ther. 2013 Aug;94(2):169-72. doi: 10.1038/clpt.2013.101. | Roden DM | 2013 |
| 34117307 | Sci Rep. 2021 Jun 11;11(1):12343. doi: 10.1038/s41598-021-90969-y. | Sukprasong R | 2021 |
| 34064668 | J Pers Med. 2021 May 11;11(5):394. doi: 10.3390/jpm11050394. | Gill PS | 2021 |
| 32602114 | Clin Pharmacol Ther. 2021 Feb;109(2):352-366. doi: 10.1002/cpt.1973. Epub 2020 Jul 22. | Botton MR | 2021 |
| 30360673 | J Int Med Res. 2018 Dec;46(12):4965-4973. doi: 10.1177/0300060518787718. Epub 2018 Oct 25. | Zheng LJ | 2018 |
| 30196751 | Pharmacogenomics. 2018 Oct;19(15):1203-1216. doi: 10.2217/pgs-2018-0097. Epub 2018 Sep 10. | Luzum JA | 2018 |
| 37448335 | Clin Transl Sci. 2023 Sep;16(9):1594-1605. doi: 10.1111/cts.13590. Epub 2023 Jul 23. | Chan CC | 2023 |
| 34880651 | Pharmgenomics Pers Med. 2021 Dec 2;14:1575-1582. doi: 10.2147/PGPM.S324612. eCollection 202 | Rytkin E | 2021 |
| 30779635 | Circ Genom Precis Med. 2019 Feb;12(2):e002441. doi: 10.1161/CIRCGEN.119.002441. | Williams AK | 2019 |
| 35652783 | Eur Heart J Qual Care Clin Outcomes. 2023 Apr 26;9(3):249-257. doi: 10.1093/ehjqcco/qcac031. | Dong OM | 2023 |
| 31260137 | Hum Mutat. 2019 Nov;40(11):e37-e51. doi: 10.1002/humu.23855. | Botton MR | 2019 |
| 34900826 | J Diabetes Metab Disord. 2021 Jul 7;20(2):1793-1805. doi: 10.1007/s40200-021-00840-0. eCollectio | Sheikhy A | 2021 |
| 31159795 | BMC Med Genomics. 2019 Jun 3;12(1):81. doi: 10.1186/s12920-019-0527-2. | Gulilat M | 2019 |
| 31162291 | Pharmacogenet Genomics. 2019 Sep;29(7):167-178. doi: 10.1097/FPC.0000000000000380. | Tshabalala S | 2019 |

| | | | |
|---|---|---|---|
| 24857912 | Int J Mol Sci. 2014 May 5;15(5):7699-710. doi: 10.3390/ijms15057699. | Jeong HE | 2014 |
| 32378381 | Croat Med J. 2020 Apr 30;61(2):147-158. doi: 10.3325/cmj.2020.61.147. | Božina N | 2020 |
| 34151428 | Clin Pharmacol Ther. 2021 Sep;110(3):714-722. doi: 10.1002/cpt.2323. Epub 2021 Jul 12. | Muhammad A | 2021 |
| 34233023 | Clin Pharmacol Ther. 2021 Oct;110(4):909-925. doi: 10.1002/cpt.2357. Epub 2021 Jul 29. | Davis BH | 2021 |
| 26606281 | BMC Med Inform Decis Mak. 2015;15 Suppl 4(Suppl 4):S3. doi: 10.1186/1472-6947-15-S4-S3. Epub 2 | Zhu Q | 2015 |
| 26430534 | J Pathol Inform. 2015 Aug 31;6:46. doi: 10.4103/2153-3539.163985. eCollection 2015. | Herr TM | 2015 |
| 28457931 | World Neurosurg. 2017 Aug;104:205-212. doi: 10.1016/j.wneu.2017.04.111. Epub 2017 Apr 27. | Moshayedi H | 2017 |
| 34501219 | J Clin Med. 2021 Aug 24;10(17):3772. doi: 10.3390/jcm10173772. | Zubiaur P | 2021 |
| 26369774 | Clin Pharmacokinet. 2016 Apr;55(4):419-36. doi: 10.1007/s40262-015-0324-9. | Maggo SD | 2016 |
| 28749586 | Clin Transl Sci. 2018 Jan;11(1):71-76. doi: 10.1111/cts.12493. Epub 2017 Jul 27. | Hicks JK | 2018 |
| 33411687 | Aging (Albany NY). 2020 Dec 19;13(3):3994-4006. doi: 10.18632/aging.202366. Epub 2020 Dec 19. | Zhou M | 2020 |
| 33437865 | Transplant Direct. 2021 Jan 7;7(2):e650. doi: 10.1097/TXD.0000000000001102. eCollection 2021 Fe | Nobakht E | 2021 |
| 38581109 | Clin Transl Sci. 2024 Apr;17(4):e13792. doi: 10.1111/cts.13792. | Biswas M | 2024 |
| 33428770 | Clin Pharmacol Ther. 2021 Jul;110(1):179-188. doi: 10.1002/cpt.2161. Epub 2021 Feb 16. | Hicks JK | 2021 |
| 29793377 | Pharmacogenomics. 2018 Jun 1;19(9):771-782. doi: 10.2217/pgs-2018-0049. Epub 2018 May 25. | Dong OM | 2018 |
| 34449608 | Diseases. 2021 Aug 12;9(3):55. doi: 10.3390/diseases9030055. | Noyes JD | 2021 |
| 24882820 | J Physiol. 2014 Jun 1;592(11):2381-8. doi: 10.1113/jphysiol.2014.272336. | Joyner MJ | 2014 |
| 27038722 | Int J Cardiol. 2016 Jun 1;212:148-50. doi: 10.1016/j.ijcard.2016.03.067. Epub 2016 Mar 18. | Chikata Y | 2016 |
| 33727661 | Sci Rep. 2021 Mar 16;11(1):5974. doi: 10.1038/s41598-021-85580-0. | Li YJ | 2021 |
| 27350664 | J Genet. 2016 Jun;95(2):231-7. doi: 10.1007/s12041-016-0618-1. | Liu X | 2016 |
| 26218263 | PLoS One. 2015 Jul 28;10(7):e0134174. doi: 10.1371/journal.pone.0134174. eCollection 2015. | Lin G | 2015 |
| 36916827 | J Clin Lab Anal. 2023 Mar;37(5):e24855. doi: 10.1002/jcla.24855. Epub 2023 Mar 14. | Huang Q | 2023 |
| 31512486 | Clin Appl Thromb Hemost. 2019 Jan-Dec;25:1076029619875520. doi: 10.1177/1076029619875520. | Ali Z | 2019 |
| 31531091 | Iran J Pharm Res. 2019 Spring;18(2):1097-1102. doi: 10.22037/ijpr.2019.1100644. | Riaz S | 2019 |
| 31171523 | BMJ. 2019 Jun 6;365:l2211. doi: 10.1136/bmj.l2211. | Wang Y | 2019 |
| 31123001 | BMJ Open. 2019 May 22;9(5):e028595. doi: 10.1136/bmjopen-2018-028595. | Zhang XG | 2019 |
| 24479687 | Curr Drug Metab. 2014 Feb;15(2):209-17. doi: 10.2174/1389200215666140130124910. | Caudle KE | 2014 |
| 30906739 | Front Med (Lausanne). 2019 Mar 8;6:38. doi: 10.3389/fmed.2019.00038. eCollection 2019. | Roses SM | 2019 |
| 25154978 | Nat Rev Cardiol. 2014 Oct;11(10):597-606. doi: 10.1038/nrcardio.2014.104. Epub 2014 Aug 26. | Levine GN | 2014 |
| 28785581 | Biomed Res Int. 2017;2017:5783719. doi: 10.1155/2017/5783719. Epub 2017 Jul 13. | Ruedlinger J | 2017 |
| 27885282 | Nat Rev Drug Discov. 2017 Jan;16(1):1. doi: 10.1038/nrd.2016.234. Epub 2016 Nov 25. | Giacomini KM | 2017 |

| | | | |
|---|---|---|---|
| 28707077 | Eur J Clin Pharmacol. 2017 Oct;73(10):1261-1269. doi: 10.1007/s00228-017-2298-z. Epub 2017 Jul 1 | Li X | 2017 |
| 28213088 | Pharmacol Ther. 2017 Jul;175:75-90. doi: 10.1016/j.pharmthera.2017.02.036. Epub 2017 Feb 14. | Zhang G | 2017 |
| 28653333 | Clin Pharmacol Ther. 2018 Feb;103(2):281-286. doi: 10.1002/cpt.780. Epub 2017 Sep 19. | Tornio A | 2018 |
| 28244804 | Pharmacogenomics. 2017 Mar;18(4):327-335. doi: 10.2217/pgs-2016-0175. Epub 2017 Feb 22. | Haga SB | 2017 |
| 29033601 | Pharmgenomics Pers Med. 2017 Sep 27;10:253-259. doi: 10.2147/PGPM.S141935. eCollection 2017 | Denisenko NP | 2017 |
| 26918067 | Curr Fungal Infect Rep. 2015 Jun;9(2):74-87. doi: 10.1007/s12281-015-0219-0. Epub 2015 Apr 16. | Moriyama B | 2015 |
| 35914768 | Annu Rev Pharmacol Toxicol. 2023 Jan 20;63:211-229. doi: 10.1146/annurev-pharmtox-051921-0927 | Castrichini M | 2023 |
| 40940774 | Cells. 2025 Sep 1;14(17):1363. doi: 10.3390/cells14171363. | Rzeczycki P | 2025 |
| 27249515 | Pharmacogenomics. 2016 Jun;17(8):853-66. doi: 10.2217/pgs-2015-0007. Epub 2016 Jun 1. | Oetjens MT | 2016 |
| 27056735 | Indian J Gastroenterol. 2016 Mar;35(2):117-22. doi: 10.1007/s12664-016-0645-0. Epub 2016 Apr 8. | Ayub A | 2016 |
| 33744207 | JACC Cardiovasc Interv. 2021 Apr 12;14(7):739-750. doi: 10.1016/j.jcin.2021.01.024. Epub 2021 Mar | Pereira NL | 2021 |
| 26150610 | BMJ Case Rep. 2015 Jul 6;2015:bcr2014205227. doi: 10.1136/bcr-2014-205227. | Droppa M | 2015 |
| 32585929 | J Clin Med. 2020 Jun 23;9(6):1963. doi: 10.3390/jcm9061963. | Numasawa Y | 2020 |
| 36072880 | Front Cardiovasc Med. 2022 Aug 22;9:925518. doi: 10.3389/fcvm.2022.925518. eCollection 2022. | Zhang M | 2022 |
| 26173871 | Expert Opin Drug Metab Toxicol. 2015;11(10):1599-617. doi: 10.1517/17425255.2015.1068757. Epu | Yang Y | 2015 |
| 25897256 | Pharmgenomics Pers Med. 2015 Feb 9;8:43-61. doi: 10.2147/PGPM.S52900. eCollection 2015. | Beitelshees AL | 2015 |
| 33033370 | Pharmacogenomics J. 2021 Apr;21(2):116-127. doi: 10.1038/s41397-020-00189-2. Epub 2020 Oct 9 | Valeria C | 2021 |
| 35365779 | Pharmacogenomics J. 2022 May;22(3):188-197. doi: 10.1038/s41397-022-00275-7. Epub 2022 Apr 1 | Jiang S | 2022 |
| 35155631 | Front Cardiovasc Med. 2022 Jan 28;9:805525. doi: 10.3389/fcvm.2022.805525. eCollection 2022. | Jourdi G | 2022 |
| 34934339 | Pharmgenomics Pers Med. 2021 Dec 14;14:1619-1628. doi: 10.2147/PGPM.S338198. eCollection 20 | Zhang J | 2021 |
| 37763139 | J Pers Med. 2023 Sep 12;13(9):1371. doi: 10.3390/jpm13091371. | Baturina O | 2023 |
| 31134829 | Cell Transplant. 2019 Aug;28(8):1039-1044. doi: 10.1177/0963689719851769. Epub 2019 May 28. | Lan H | 2019 |
| 24619595 | Am J Med Genet C Semin Med Genet. 2014 Mar;166C(1):45-55. doi: 10.1002/ajmg.c.31391. Epub 201 | Hoffman JM | 2014 |
| 26756170 | Clin Pharmacol Ther. 2016 Apr;99(4):401-4. doi: 10.1002/cpt.333. Epub 2016 Feb 15. | O'Donnell PH | 2016 |
| 26592190 | Nat Rev Nephrol. 2016 Feb;12(2):110-22. doi: 10.1038/nrneph.2015.176. Epub 2015 Nov 23. | Cooper-DeHoff RM | 2016 |
| 34242414 | Pharmacotherapy. 2021 Dec;41(12):970-977. doi: 10.1002/phar.2611. Epub 2021 Jul 21. | Wood B | 2021 |
| 34501440 | J Clin Med. 2021 Sep 3;10(17):3992. doi: 10.3390/jcm10173992. | Olie RH | 2021 |
| 28222641 | J Int Med Res. 2017 Feb;45(1):134-146. doi: 10.1177/0300060516677190. Epub 2016 Dec 22. | Tam CC | 2017 |
| 28245774 | Curr Clin Pharmacol. 2017;12(1):11-17. doi: 10.2174/1574884712666170227154654. | Duconge J | 2017 |
| 28994452 | Clin Pharmacol Ther. 2018 Apr;103(4):599-618. doi: 10.1002/cpt.762. Epub 2017 Oct 10. | Bank PCD | 2018 |
| 29102571 | JACC Cardiovasc Interv. 2018 Jan 22;11(2):181-191. doi: 10.1016/j.jcin.2017.07.022. Epub 2017 Nov | Cavallari LH | 2018 |

| | | | |
|---|---|---|---|
| 33447439 | J Thorac Dis. 2020 Dec;12(12):7501-7503. doi: 10.21037/jtd-20-3066. | Kim HK | 2020 |
| 33188450 | Eur J Clin Pharmacol. 2021 Apr;77(4):643-650. doi: 10.1007/s00228-020-03043-3. Epub 2020 Nov 13 | Kauppila M | 2021 |
| 33430289 | J Pers Med. 2021 Jan 7;11(1):33. doi: 10.3390/jpm11010033. | Han N | 2021 |
| 32077359 | Pharmacogenomics. 2020 Apr;21(6):375-386. doi: 10.2217/pgs-2020-0007. Epub 2020 Feb 20. | Aquilante CL | 2020 |
| 33528719 | Cardiovasc Drugs Ther. 2021 Jun;35(3):663-676. doi: 10.1007/s10557-021-07149-3. Epub 2021 Feb | Magavern EF | 2021 |
| 33234119 | BMC Med. 2020 Nov 25;18(1):367. doi: 10.1186/s12916-020-01827-z. | Turner RM | 2020 |
| 26108379 | J Manag Care Spec Pharm. 2015 Jul;21(7):552-7. doi: 10.18553/jmcp.2015.21.7.552. | Johnson SG | 2015 |
| 32644829 | J Int Med Res. 2020 Jul;48(7):300060520934657. doi: 10.1177/0300060520934657. | Bai Y | 2020 |
| 36561777 | Front Cardiovasc Med. 2022 Dec 6;9:1023004. doi: 10.3389/fcvm.2022.1023004. eCollection 2022. | Yao H | 2022 |
| 36581799 | BMC Cardiovasc Disord. 2022 Dec 30;22(1):575. doi: 10.1186/s12872-022-02988-w. | Zhang YJ | 2022 |
| 36707467 | Neurosurg Rev. 2023 Jan 28;46(1):42. doi: 10.1007/s10143-023-01952-2. | Zhang Y | 2023 |
| 35250581 | Front Pharmacol. 2022 Feb 18;13:835136. doi: 10.3389/fphar.2022.835136. eCollection 2022. | Biswas M | 2022 |
| 31527945 | Mo Med. 2019 May-Jun;116(3):217-225. | Eissenberg JC | 2019 |
| 30564130 | Front Pharmacol. 2018 Dec 4;9:1436. doi: 10.3389/fphar.2018.01436. eCollection 2018. | Zhou S | 2018 |
| 31173123 | JAMA Netw Open. 2019 Jun 5;2(6):e195345. doi: 10.1001/jamanetworkopen.2019.5345. | Chanfreau-Coffinier C | 2019 |
| 30936195 | Cold Spring Harb Mol Case Stud. 2019 Apr 1;5(2):a003731. doi: 10.1101/mcs.a003731. Print 2019 Ap | Bonney PA | 2019 |
| 34713722 | J Am Heart Assoc. 2021 Nov 2;10(21):e021129. doi: 10.1161/JAHA.121.021129. Epub 2021 Oct 29. | Liu YL | 2021 |
| 29844222 | Cold Spring Harb Perspect Med. 2019 Feb 1;9(2):a033027. doi: 10.1101/cshperspect.a033027. | Schwarz UI | 2019 |
| 37287029 | Hum Genomics. 2023 Jun 7;17(1):51. doi: 10.1186/s40246-023-00495-3. | Koufaki MI | 2023 |
| 27864204 | Am J Health Syst Pharm. 2016 Dec 1;73(23):1967-1976. doi: 10.2146/ajhp160030. | Hicks JK | 2016 |
| 27886818 | Interv Cardiol Clin. 2017 Jan;6(1):141-149. doi: 10.1016/j.iccl.2016.08.010. | Cavallari LH | 2017 |
| 28306618 | Pharmacogenet Genomics. 2017 May;27(5):190-196. doi: 10.1097/FPC.0000000000000277. | Hamadeh IS | 2017 |
| 28900583 | Hawaii J Med Public Health. 2017 Sep;76(9):265-269. | Ciarleglio AE | 2017 |
| 25187485 | Drug Metab Dispos. 2014 Nov;42(11):1971-7. doi: 10.1124/dmd.114.060285. Epub 2014 Sep 3. | Zhu AZ | 2014 |
| 39868895 | Clin Transl Sci. 2025 Feb;18(2):e70143. doi: 10.1111/cts.70143. | Chia CY | 2025 |
| 39991772 | Int J Med Sci. 2025 Jan 21;22(4):903-919. doi: 10.7150/ijms.101219. eCollection 2025. | Lee CL | 2025 |
| 29095091 | Pharmacogenomics. 2017 Nov;18(16):1541-1550. doi: 10.2217/pgs-2017-0076. Epub 2017 Nov 2. | Martin A | 2017 |
| 35912831 | Expert Rev Clin Pharmacol. 2022 Jul;15(7):811-825. doi: 10.1080/17512433.2022.2108401. Epub 20 | McDermott JH | 2022 |
| 25732742 | Curr Atheroscler Rep. 2015 May;17(5):501. doi: 10.1007/s11883-015-0501-1. | Quatromoni N | 2015 |
| 32764090 | BMJ Open. 2020 Aug 6;10(8):e038936. doi: 10.1136/bmjopen-2020-038936. | Hernandez-Suarez DF | 2020 |
| 33915807 | Molecules. 2021 Apr 1;26(7):1987. doi: 10.3390/molecules26071987. | Akkaif MA | 2021 |

| | | | |
|---|---|---|---|
| 26019129 | J Am Heart Assoc. 2015 May 27;4(6):e001652. doi: 10.1161/JAHA.114.001652. | McDonough CW | 2015 |
| 32623598 | Cardiovasc Drugs Ther. 2021 Jun;35(3):549-559. doi: 10.1007/s10557-020-06988-w. | Morales-Rosado JA | 2021 |
| 27450232 | Eur J Clin Pharmacol. 2016 Oct;72(10):1195-1204. doi: 10.1007/s00228-016-2094-1. Epub 2016 Jul | Zhao Z | 2016 |
| 33995083 | Front Pharmacol. 2021 Apr 29;12:660639. doi: 10.3389/fphar.2021.660639. eCollection 2021. | Mejía-Abril G | 2021 |
| 25957826 | J Biomed Inform. 2015 Jun;55:249-59. doi: 10.1016/j.jbi.2015.04.011. Epub 2015 May 7. | Overby CL | 2015 |
| 26177117 | CNS Neurosci Ther. 2015 Sep;21(9):692-7. doi: 10.1111/cns.12426. Epub 2015 Jul 15. | Han Y | 2015 |
| 35249906 | J Atheroscler Thromb. 2023 Jan 1;30(1):39-55. doi: 10.5551/jat.63369. Epub 2022 Mar 5. | Fukuma K | 2023 |
| 30498685 | Cardiovasc Diagn Ther. 2018 Oct;8(5):610-620. doi: 10.21037/cdt.2018.05.06. | Strisciuglio T | 2018 |
| 31772608 | Cardiovasc Ther. 2019 Jul 18;2019:3470145. doi: 10.1155/2019/3470145. eCollection 2019. | Peng W | 2019 |
| 31041128 | Neurol Clin Pract. 2019 Apr;9(2):140-144. doi: 10.1212/CPJ.0000000000000584. | Lyerly MJ | 2019 |
| 32320492 | Br J Clin Pharmacol. 2020 Aug;86(8):1489-1498. doi: 10.1111/bcp.14317. Epub 2020 Apr 28. | Yoon HY | 2020 |
| 35082514 | Pharmgenomics Pers Med. 2022 Jan 20;15:29-43. doi: 10.2147/PGPM.S338287. eCollection 2022. | Yuan D | 2022 |
| 32176040 | Medicine (Baltimore). 2020 Mar;99(11):e19143. doi: 10.1097/MD.0000000000019143. | Liu G | 2020 |
| 32100936 | Clin Transl Sci. 2020 Sep;13(5):861-870. doi: 10.1111/cts.12771. Epub 2020 Apr 13. | Lo C | 2020 |
| 31141104 | JAMA Cardiol. 2019 Jul 1;4(7):680-684. doi: 10.1001/jamacardio.2019.1510. | Povsic TJ | 2019 |
| 29335863 | Chin J Integr Med. 2019 May;25(5):395-400. doi: 10.1007/s11655-017-2551-4. Epub 2018 Jan 15. | Chen H | 2019 |
| 29610748 | Ann Transl Med. 2018 Feb;6(3):56. doi: 10.21037/atm.2017.11.36. | Han X | 2018 |
| 29615454 | Circ Genom Precis Med. 2018 Apr;11(4):e002069. doi: 10.1161/CIRCGEN.117.002069. | Lee CR | 2018 |
| 34272705 | Methods Mol Biol. 2021;2342:481-550. doi: 10.1007/978-1-0716-1554-6_17. | Lin YS | 2021 |
| 34461749 | Circ Genom Precis Med. 2021 Oct;14(5):e003178. doi: 10.1161/CIRCGEN.121.003178. Epub 2021 A | Clarke SL | 2021 |
| 25220280 | Pharmacotherapy. 2014 Oct;34(10):1102-12. doi: 10.1002/phar.1481. Epub 2014 Sep 15. | Owusu-Obeng A | 2014 |
| 34177775 | Front Neurol. 2021 Jun 10;12:667234. doi: 10.3389/fneur.2021.667234. eCollection 2021. | Alhazzani A | 2021 |
| 25521360 | Pharmacogenomics. 2014 Dec;15(16):2025-48. doi: 10.2217/pgs.14.144. | Chhibber A | 2014 |
| 26976545 | Circ Cardiovasc Genet. 2016 Apr;9(2):193-202. doi: 10.1161/HCG.0000000000000029. Epub 2016 N | Hall JL | 2016 |
| 27864205 | Am J Health Syst Pharm. 2016 Dec 1;73(23):1977-1985. doi: 10.2146/ajhp150977. | Caudle KE | 2016 |
| 26445541 | Ther Clin Risk Manag. 2015 Sep 23;11:1421-7. doi: 10.2147/TCRM.S83293. eCollection 2015. | Saab YB | 2015 |
| 28125086 | Genet Med. 2017 Aug;19(8):890-899. doi: 10.1038/gim.2016.209. Epub 2017 Jan 26. | Lynch JA | 2017 |
| 29181084 | Per Med. 2017 Sep;14(5):383-388. doi: 10.2217/pme-2017-0022. Epub 2017 Sep 1. | Mukherjee C | 2017 |
| 33428771 | Int J Cancer. 2021 Jun 1;148(11):2799-2806. doi: 10.1002/ijc.33469. Epub 2021 Jan 19. | Krens SD | 2021 |
| 40118816 | Bioanalysis. 2025 Mar;17(6):399-411. doi: 10.1080/17576180.2025.2481019. Epub 2025 Mar 21. | Zhang XT | 2025 |
| 33198260 | Genes (Basel). 2020 Nov 12;11(11):1337. doi: 10.3390/genes11111337. | Rollinson V | 2020 |

| | | | |
|---|---|---|---|
| 40933569 | Front Med (Lausanne). 2025 Aug 26;12:1660889. doi: 10.3389/fmed.2025.1660889. eCollection 202 | Zhuang H | 2025 |
| 39302533 | Cardiovasc Interv Ther. 2024 Oct;39(4):335-375. doi: 10.1007/s12928-024-01036-y. Epub 2024 Sep | Ozaki Y | 2024 |
| 33953382 | Nat Rev Cardiol. 2021 Sep;18(9):649-665. doi: 10.1038/s41569-021-00549-w. Epub 2021 May 5. | Duarte JD | 2021 |
| 32678355 | Genet Med. 2020 Nov;22(11):1898-1902. doi: 10.1038/s41436-020-0894-2. Epub 2020 Jul 17. | Beitelshees AL | 2020 |
| 31562822 | Clin Pharmacol Ther. 2020 Jan;107(1):171-175. doi: 10.1002/cpt.1651. Epub 2019 Nov 5. | Relling MV | 2020 |
| 30191366 | J Neural Transm (Vienna). 2019 Jan;126(1):5-18. doi: 10.1007/s00702-018-1922-0. Epub 2018 Sep 6. | Mostafa S | 2019 |
| 31367173 | Ethn Dis. 2019 Jul 18;29(3):517-524. doi: 10.18865/ed.29.3.517. eCollection 2019 Summer. | Mamun A | 2019 |
| 35869255 | Pharmacogenomics J. 2022 Dec;22(5-6):264-275. doi: 10.1038/s41397-022-00285-5. Epub 2022 Jul | Albalwy F | 2022 |
| 34824386 | Pharmacogenomics J. 2022 Mar;22(2):100-108. doi: 10.1038/s41397-021-00262-4. Epub 2021 Nov 2 | Jaya Shankar A | 2022 |
| 35647265 | AIMS Mol Sci. 2022;9(2):66-78. doi: 10.3934/molsci.2022004. Epub 2022 Apr 28. | Tekeste R | 2022 |
| 29848980 | Int J Environ Res Public Health. 2018 May 30;15(6):1115. doi: 10.3390/ijerph15061115. | Hernandez-Suarez DF | 2018 |
| 37602077 | Cureus. 2023 Jul 19;15(7):e42169. doi: 10.7759/cureus.42169. eCollection 2023 Jul. | Amarapalli J | 2023 |
| 30896564 | Chin Med J (Engl). 2019 May 5;132(9):1053-1062. doi: 10.1097/CM9.0000000000000210. | Ma Q | 2019 |
| 29655499 | Am J Kidney Dis. 2018 Oct;72(4):569-581. doi: 10.1053/j.ajkd.2018.02.351. Epub 2018 Apr 11. | Franceschini N | 2018 |
| 24710841 | J Clin Pharmacol. 2014 Aug;54(8):865-73. doi: 10.1002/jcph.293. Epub 2014 Apr 7. | Horenstein RB | 2014 |
| 29922082 | Pharmgenomics Pers Med. 2018 Jun 8;11:95-106. doi: 10.2147/PGPM.S165805. eCollection 2018. | Hernandez-Suarez DF | 2018 |
| 34263311 | J Appl Lab Med. 2021 Nov 1;6(6):1505-1516. doi: 10.1093/jalm/jfab056. | Tang NY | 2021 |
| 25206309 | Pharmgenomics Pers Med. 2014 Aug 13;7:227-40. doi: 10.2147/PGPM.S48887. eCollection 2014. | Abul-Husn NS | 2014 |
| 34431601 | Clin Transl Sci. 2021 Nov;14(6):2532-2543. doi: 10.1111/cts.13121. Epub 2021 Aug 25. | Grace C | 2021 |
| 28690699 | Pharm Pract (Granada). 2017 Apr-Jun;15(2):946. doi: 10.18549/PharmPract.2017.02.946. Epub 2017 | Johnson SG | 2017 |
| 28950720 | Exp Biol Med (Maywood). 2018 Feb;243(3):291-299. doi: 10.1177/1535370217733425. Epub 2017 Se | Carr DF | 2018 |
| 28819726 | Clin Pharmacokinet. 2018 Jun;57(6):739-748. doi: 10.1007/s40262-017-0592-7. | Peigné S | 2018 |
| 28421156 | Cardiol Res Pract. 2017;2017:8062796. doi: 10.1155/2017/8062796. Epub 2017 Mar 21. | Amin AM | 2017 |
| 33611730 | Transl Stroke Res. 2022 Feb;13(1):46-55. doi: 10.1007/s12975-021-00896-3. Epub 2021 Feb 21. | Patel PD | 2022 |
| 38253063 | Am J Health Syst Pharm. 2024 Jun 11;81(12):555-562. doi: 10.1093/ajhp/zxae008. | Aquilante CL | 2024 |
| 39148087 | BMC Med. 2024 Aug 15;22(1):335. doi: 10.1186/s12916-024-03549-y. | Kim JH | 2024 |
| 30745309 | Drug Metab Dispos. 2019 Apr;47(4):425-435. doi: 10.1124/dmd.118.084269. Epub 2019 Feb 11. | Devarajan S | 2019 |
| 29280137 | Clin Pharmacol Ther. 2018 Oct;104(4):664-674. doi: 10.1002/cpt.1006. Epub 2018 Jan 30. | Empey PE | 2018 |
| 40441245 | Medicine (Baltimore). 2025 May 30;104(22):e42551. doi: 10.1097/MD.0000000000042551. | Cheng B | 2025 |
| 38884958 | Pharmacogenomics. 2024;25(7):293-298. doi: 10.1080/14622416.2024.2355862. Epub 2024 Jun 6. | Park JJ | 2024 |
| 38752549 | Health Econ Policy Law. 2024 Oct;19(4):446-458. doi: 10.1017/S1744133124000070. Epub 2024 Ma | Dixon P | 2024 |

| | | | |
|---|---|---|---|
| 40926880 | Res Pract Thromb Haemost. 2025 Aug 7;9(6):102997. doi: 10.1016/j.rpth.2025.102997. eCollection 2 | Dang D | 2025 |
| 40667755 | ACS Sens. 2025 Sep 26;10(9):6819-6827. doi: 10.1021/acssensors.5c01577. Epub 2025 Jul 16. | Serapinas S | 2025 |
| 36149409 | Clin Pharmacol Ther. 2022 Dec;112(6):1318-1328. doi: 10.1002/cpt.2754. Epub 2022 Oct 9. | Morris SA | 2022 |
| 33917299 | Biology (Basel). 2021 Apr 6;10(4):300. doi: 10.3390/biology10040300. | Miftahussurur M | 2021 |
| 34110908 | Circ Res. 2021 Jun 11;128(12):1973-1987. doi: 10.1161/CIRCRESAHA.121.318259. Epub 2021 Jun 1 | Fleming MR | 2021 |
| 33805706 | J Pers Med. 2021 Mar 13;11(3):204. doi: 10.3390/jpm11030204. | Zubiaur P | 2021 |
| 34065778 | J Pers Med. 2021 May 12;11(5):400. doi: 10.3390/jpm11050400. | Angulo-Aguado M | 2021 |
| 38409912 | Brain Behav. 2024 Feb;14(2):e3439. doi: 10.1002/brb3.3439. | Wang K | 2024 |
| 27311679 | Clin Pharmacol Ther. 2016 Oct;100(4):380-8. doi: 10.1002/cpt.411. Epub 2016 Aug 18. | Yang W | 2016 |
| 31858263 | Int J Legal Med. 2020 Mar;134(2):433-439. doi: 10.1007/s00414-019-02234-7. Epub 2019 Dec 20. | Skadrić I | 2020 |
| 31453360 | MDM Policy Pract. 2019 Aug 17;4(2):2381468319864337. doi: 10.1177/2381468319864337. eColle | Shi Y | 2019 |
| 30073432 | Eur J Clin Pharmacol. 2018 Dec;74(12):1567-1574. doi: 10.1007/s00228-018-2530-5. Epub 2018 Au | Chouchene S | 2018 |
| 30697355 | Eur Cardiol. 2018 Dec;13(2):112-114. doi: 10.15420/ecr.2018.13.2.EO2. | Su-Yin DT | 2018 |
| 32460360 | Clin Pharmacol Ther. 2020 Sep;108(3):557-565. doi: 10.1002/cpt.1912. Epub 2020 Jul 6. | Marrero RJ | 2020 |
| 32081407 | Semin Perinatol. 2020 Apr;44(3):151222. doi: 10.1016/j.semperi.2020.151222. Epub 2020 Jan 25. | Betcher HK | 2020 |
| 24253661 | Clin Pharmacol Ther. 2014 Apr;95(4):423-31. doi: 10.1038/clpt.2013.229. Epub 2013 Nov 19. | Van Driest SL | 2014 |
| 29382029 | Medicine (Baltimore). 2017 Nov;96(47):e8920. doi: 10.1097/MD.0000000000008920. | Ding L | 2017 |
| 31961467 | Clin Transl Sci. 2020 May;13(3):618-627. doi: 10.1111/cts.12748. Epub 2020 Feb 12. | Smith DM | 2020 |
| 24616371 | Am J Med Genet C Semin Med Genet. 2014 Mar;166C(1):56-67. doi: 10.1002/ajmg.c.31390. Epub 20 | Weitzel KW | 2014 |
| 24616408 | Am J Med Genet C Semin Med Genet. 2014 Mar;166C(1):76-84. doi: 10.1002/ajmg.c.31396. Epub 20 | Shuldiner AR | 2014 |
| 34357562 | Mol Diagn Ther. 2021 Nov;25(6):735-755. doi: 10.1007/s40291-021-00549-z. Epub 2021 Aug 6. | Hirata TDC | 2021 |
| 28070879 | Eur J Drug Metab Pharmacokinet. 2017 Oct;42(5):745-756. doi: 10.1007/s13318-016-0399-1. | Elewa H | 2017 |
| 34564559 | Pharmacy (Basel). 2021 Sep 6;9(3):152. doi: 10.3390/pharmacy9030152. | Stäuble CK | 2021 |
| 33262635 | Pharmgenomics Pers Med. 2020 Nov 23;13:645-653. doi: 10.2147/PGPM.S279719. eCollection 2020 | Sun J | 2020 |
| 40109368 | Front Pharmacol. 2025 Mar 5;15:1494482. doi: 10.3389/fphar.2024.1494482. eCollection 2024. | Pérez-Duval E | 2025 |
| 39960900 | Medicine (Baltimore). 2025 Feb 14;104(7):e41368. doi: 10.1097/MD.0000000000041368. | Wei X | 2025 |
| 35134542 | J Mol Diagn. 2022 Apr;24(4):337-350. doi: 10.1016/j.jmoldx.2021.12.011. Epub 2022 Feb 5. | Gaedigk A | 2022 |
| 40593689 | NPJ Genom Med. 2025 Jul 1;10(1):50. doi: 10.1038/s41525-025-00508-1. | Lucas Beckett IA | 2025 |
